# Dose response of the 16p11.2 distal copy number variant on intracranial volume and basal ganglia

**DOI:** 10.1038/s41380-018-0118-1

**Published:** 2018-10-03

**Authors:** Ida E Sønderby, Ómar Gústafsson, Nhat Trung Doan, Derrek P Hibar, Sandra Martin-Brevet, Abdel Abdellaoui, David Ames, Katrin Amunts, Michael Andersson, Nicola J Armstrong, Manon Bernard, Nicholas Blackburn, John Blangero, Dorret I Boomsma, Janita Bralten, Hans-Richard Brattbak, Henry Brodaty, Rachel M Brouwer, Robin Bülow, Vince Calhoun, Svenja Caspers, Gianpiero Cavalleri, Chi-Hua Chen, Sven Cichon, Simone Ciufolini, Aiden Corvin, Benedicto Crespo-Facorro, Joanne E Curran, Anders M Dale, Shareefa Dalvie, Paola Dazzan, Eco J C de Geus, Greig I. de Zubicaray, Sonja M. C. de Zwarte, Norman Delanty, Anouk den Braber, Sylvane Desrivières, Gary Donohoe, Bogdan Draganski, Stefan Ehrlich, Thomas Espeseth, Simon E Fisher, Barbara Franke, Vincent Frouin, Masaki Fukunaga, Thomas Gareau, David C Glahn, Hans Grabe, Nynke A. Groenewold, Jan Haavik, Asta Håberg, Ryota Hashimoto, Jayne Y Hehir-Kwa, Andreas Heinz, Manon H. J. Hillegers, Per Hoffmann, Laurena Holleran, Jouke-Jan Hottenga, Hilleke E Hulshoff, Masashi Ikeda, Neda Jahanshad, Terry Jernigan, Christiane Jockwitz, Stefan Johansson, Gudrun A Jonsdottir, Erik G Jönsson, Rene Kahn, Tobias Kaufmann, Sinead Kelly, Masataka Kikuchi, Emma E M Knowles, Knut K Kolskår, John B Kwok, Stephanie Le Hellard, Costin Leu, Jingyu Liu, Astri J Lundervold, Arvid Lundervold, Nicholas G. Martin, Karen Mather, Samuel R. Mathias, Mark McCormack, Katie L. McMahon, Allan McRae, Yuri Milaneschi, Clara Moreau, Derek Morris, David Mothersill, Thomas W Mühleisen, Robin Murray, Jan E Nordvik, Lars Nyberg, Loes M Olde Loohuis, Roel Ophoff, Tomas Paus, Zdenka Pausova, Brenda Penninx, Juan M Peralta, Bruce Pike, Carlos Prieto, Sara Pudas, Erin Quinlan, Daniel S Quintana, Céline S Reinbold, Tiago Reis Marques, Alexandre Reymond, Genevieve Richard, Borja Rodriguez-Herreros, Roberto Roiz-Santiañez, Jarek Rokicki, James Rucker, Perminder Sachdev, Anne-Marthe Sanders, Sigrid B Sando, Lianne Schmaal, Peter R Schofield, Andrew J. Schork, Gunter Schumann, Jean Shin, Elena Shumskaya, Sanjay Sisodiya, Vidar M Steen, Dan J Stein, Stacy Steinberg, Lachlan Strike, Alexander Teumer, Anbu Thalamuthu, Diana Tordesillas-Gutierrez, Jessica Turner, Torill Ueland, Anne Uhlmann, Magnus O. Ulfarsson, Dennis van ’t Ent, Dennis van der Meer, Neeltje E. M. van Haren, Anja Vaskinn, Evangelos Vassos, G. Bragi Walters, Yunpeng Wang, Wei Wen, Christopher D Whelan, Katharina Wittfeld, Margie Wright, Hidenaga Yamamori, Tetyana Zayats, Ingrid Agartz, Lars T Westlye, Sébastien Jacquemont, Srdjan Djurovic, Hreinn Stefánsson, Kári Stefánsson, Paul Thompson, Ole A. Andreassen, for the 16p11.2 European Consortium, for the ENIGMA-CNV working group

**Affiliations:** 1 NORMENT, K.G. Jebsen Centre for Psychosis Research, Institute of Clinical Medicine, University of Oslo and Division of Mental Health and Addiction, Oslo University Hospital, Oslo, Norway; 2 deCODE Genetics/Amgen, Reykjavik, Iceland; 3 Imaging Genetics Center, Mark and Mary Stevens Neuroimaging and Informatics Institute, Keck School of Medicine of the University of Southern California, Marina del Rey, USA; 4 Janssen Research and Development, La Jolla, CA USA; 5 Service of Medical Genetics, Centre Hospitalier Universitaire Vaudois and University of Lausanne, Rue du Bugnon 46, 1011 Lausanne, Switzerland; 6 Biological Psychology, Vrije Universiteit Amsterdam, van Boechorststraat 1, 1081 BT Amsterdam, The Netherlands; 7 Department of Psychiatry, Academic Medical Center, Amsterdam, The Netherlands; 8 National Ageing Research Institute, Melbourne, Australia; 9 Academic Unit for Psychiatry of Old Age, University of Melbourne, Melbourne, Australia; 10 Institute of Neuroscience and Medicine (INM-1), Research Centre Juelich, Wilhelm-Johnen-Str., 52425 Juelich, Germany; 11 C. and O. Vogt Institute for Brain Research, Medical Faculty, University of Dusseldorf, Merowingerplatz 1A, 40225 Dusseldorf, Germany; 12 JARA-BRAIN, Juelich-Aachen Research Alliance, Wilhelm-Johnen-Str., 52425 Juelich, Germany; 13 Umeå Center for Functional Brain Imaging (UFBI), Umeå University, 90187 Umeå, Sweden; 14 Mathematics and Statistics, Murdoch University, Perth, Australia; 15 The Hospital for Sick Children, University of Toronto, Toronto, M5G 1X8 Canada; 16 South Texas Diabetes and Obesity Institute, Department of Human Genetics, School of Medicine, University of Texas Rio Grande Valley, One West University Blvd., 78520 Brownsville, TX USA; 17 Netherlands Twin Register, Vrije Universiteit, van der Boechorststraat 1, 1081BT Amsterdam, Netherlands; 18 Department of Human Genetics, Radboud University Medical Center, Nijmegen, The Netherlands; 19 Department of Clinical Science, University of Bergen, Bergen, Norway; 20 Center for Medical Genetics and Molecular Medicine, Haukeland University Hospital, Bergen, Norway; 21 Centre for Healthy Brain Ageing and Dementia Collaborative Research Centre, UNSW, Sydney, Australia; 22 Department of Psychiatry, Brain Center Rudolf Magnus, University Medical Center Utrecht, Utrecht, The Netherlands; 23 Department of Diagnostic Radiology and Neuroradiology, University Medicine Greifswald, Greifswald, Germany; 24 The Mind Research Network, The University of New Mexico, Albuquerque, NM Mexico; 25 The Royal College of Surgeons in Ireland, 123 St Stephen’s Green, Dublin 2, Ireland; 26 Department of Radiology, University of California San Diego, La Jolla, USA; 27 Center for Multimodal Imaging and Genetics, University of California San Diego, La Jolla, USA; 28 Institute of Neuroscience and Medicine (INM-1), Structural and Functional Organisation of the Brain, Genomic Imaging, Research Centre Juelich, Leo-Brandt-Strasse 5, 52425 Jülich, Germany; 29 Human Genomics Research Group, Department of Biomedicine, University of Basel, Hebelstrasse 20, 4031 Basel, Switzerland; 30 Institute of Medical Genetics and Pathology, University Hospital Basel, Schönbeinstrasse 40, 4031 Basel, Switzerland; 31 Psychosis Studies, Insitute of Psychiatry, Psychology and Neuroscience, King’s College London, 16 De Crespingy Park, SE5 8AF London, United Kingdom; 32 Neuropsychiatric Genetics Research Group, Discipline of Psychiatry, School of Medicine, Trinity College Dublin, Dublin 2, Ireland; 33 Department of Medicine and Psychiatry, University Hospital Marqués de Valdecilla, School of Medicine, University of Cantabria-IDIVAL, 39008 Santander, Spain; 34 CIBERSAM (Centro Investigación Biomédica en Red Salud Mental), Santander, 39011 Spain; 35 Department of Psychiatry and Mental Health, Anzio Road, 7925 Cape Town, South Africa; 36 Department of Psychosis Studies, Institute of Psychiatry, Psychology and Neuroscience, King’s College London, De Crespigny Park, SE5 8AF London, United Kingdom; 37 National Institute for Health Research (NIHR) Mental Health Biomedical Research Centre at South London and Maudsley NHS Foundation Trust and King’s College London, London, United Kingdom; 38 Department of Biological Psychology, Behavioral and Movement Sciences, Vrije Universiteit, van der Boechorststraat 1, 1081 BT Amsterdam, Netherlands; 39 Amsterdam Neuroscience, VU University medical center, van der Boechorststraat 1, 1081 BT Amsterdam, NH Netherlands; 40 Faculty of Health and Institute of Health and Biomedical Innovation, Queensland University of Technology, Brisbane, Queensland Australia; 41 Imaging of Dementia and Aging (IDeA) Laboratory, Department of Neurology and Center for Neuroscience, University of California at Davis, 4860 Y Street, Suite 3700, Sacramento, California 95817 USA; 42 Alzheimer Center and Department of Neurology, VU University Medical Center, De Boelelaan 1105, 1081HV Amsterdam, Netherlands; 43 Medical Research Council – Social, Genetic and Developmental Psychiatry Centre, Institute of Psychiatry, Psychology & Neuroscience, King’s College London, London, United Kingdom; 44 Cognitive Genetics and Cognitive Therapy Group, Neuroimaging, Cognition & Genomics Centre (NICOG) & NCBES Galway Neuroscience Centre, School of Psychology and Discipline of Biochemistry, National University of Ireland Galway, H91 TK33 Galway, Ireland; 45 Neuropsychiatric Genetics Research Group, Department of Psychiatry and Trinity College Institute of Psychiatry, Trinity College Dublin, Dublin 8, Ireland; 46 LREN - Département des neurosciences cliniques, Centre Hospitalier Universitaire Vaudois and University of Lausanne, Lausanne, Switzerland; 47 Max-Planck-Institute for Human Cognitive and Brain Sciences, Leipzig, Germany; 48 Division of Psychological and Social Medicine and Developmental Neurosciences, Faculty of Medicine, TU Dresden, 01307 Dresden, Germany; 49 Department of Psychiatry, Massachusetts General Hospital, Boston, Massachusetts, 02114 USA; 50 Martinos Center for Biomedical Imaging, Massachusetts General Hospital, Charlestown, Massachusetts 02129 USA; 51 Department of Psychology, University of Oslo, Oslo, Norway; 52 Language and Genetics Department, Max Planck Institute for Psycholinguistics, Wundtlaan 1, 6525 XD Nijmegen, Netherlands; 53 Donders Institute for Brain, Cognition and Behaviour, Radboud University, Nijmegen, Netherlands; 54 Department of Psychiatry, Radboud University Medical Center, Nijmegen, The Netherlands; 55 NeuroSpin, CEA, Université Paris-Saclay, F-91191 Gif-sur-Yvette, France; 56 Division of Cerebral Integration, National Institute for Physiological Sciences, Aichi, Japan; 57 Yale University School of Medicine, 40 Temple Street, Suite 6E, 6511 New Haven, Vaud USA; 58 Olin Neuropsychiatric Research Center, Institute of Living, Hartford Hospital, 300 George Street, 6106 Hartford, CT USA; 59 Department of Psychiatry und Psychotherapy, University Medicine Greifswald, Greifswald, Germany; 60 K.G. Jebsen Centre for Neuropsychiatric Disorders, University of Bergen, Bergen, Norway; 61 Department of Neuroscience, Faculty of Medicine, Norwegian University of Science and Technology, Trondheim, Norway; 62 Molecular Research Center for Children’s Mental Development, United Graduate School of Child Development, Osaka University, Suita, Osaka Japan; 63 Princess Máxima Center for Pediatric Oncology, Lundlaan 6, 3584 EA Utrecht, The Netherlands; 64 Dept. of Psychiatry and Psychotherapie, Charite, Humboldt University, Chariteplatz 1, 10017 Berlin, Germany; 65 Child and adolescent Psychiatry / Psychology, Erasmus medical center-Sophia’s Childerens hospitaal, Rotterdam, Wytemaweg 8, 3000 CB Rotterdam, The Netherlands; 66 Institute of Human Genetics, University of Bonn, Sigmund-Freud-Str. 25, 53127 Bonn, Germany; 67 The Centre for Neuroimaging & Cognitive Genomics (NICOG) and NCBES Galway Neuroscience Centre, National University of Ireland Galway, Galway, Ireland; 68 Department of Psychiatry, Fujita Health University School of Medicine, Toyoake, Japan; 69 Center for Human Development, University of California San Diego, San Diego, CA USA; 70 Department of Psychiatry, Psychotherapy and Psychosomatics, RWTH Aachen University, Medical Faculty, Pauwelsstraße 30, 52074 Aachen, Germany; 71 Department of Clinical Neuroscience, Centre for Psychiatric Research, Karolinska Institutet, Karolinska University Hospital Solna, R5:00, SE-17176 Stockholm, Sweden; 72 Department of Genome Informatics, Graduate School of Medicine, Osaka University, 2-2, Yamadaoka, Suita, Osaka 565-0871 Japan; 73 Department of Psychiatry, Yale University, 40 Temple Street, 6515 New Haven, CT USA; 74 Sunnaas Rehabilitation Hospital HT, Nesodden, Norway; 75 Brain and Mind Centre, University of Sydney, Sydney, Australia; 76 NORMENT - KG Jebsen Centre, Department of Clinical Science, University of Bergen, Jonas Lies veg 87, 5021 Bergen, Norway; 77 Dr. Einar Martens Research Group for Biological Psychiatry, Center for Medical Genetics and Molecular Medicine, Haukeland University Hospital, Jonas Lies veg 87, 5021 Bergen, Norway; 78 Genomic Medicine Institute, Lerner Research Institute, Cleveland Clinic, Cleveland, OH USA; 79 Institute of Neurology, University College London, London, United Kingdom; 80 The Mind Research Network, 1101 Yale Blvd., 87106 Albuquerque, CT USA; 81 Dept. of Electrical and Computer Engineering, University of New Mexico, 87131 Albuquerque, New Mexico USA; 82 Department of Biological and Medical Psychology, Jonas Lies vei 91, N-5009 Bergen, Norway; 83 Department of Biomedicine, University of Bergen, 5009 Bergen, Norway; 84 QIMR Berghofer Medical Research Institute, Brisbane, Queensland Australia; 85 Centre for Healthy Brain Ageing, School of Psychiatry, University of New South Wales, Sydney, New South Wales 2052 Australia; 86 Molecular and Cellular Therapeutics, Royal College of Surgeons in Ireland, 123 St. Stephens Green, D02 YN77 Dublin, Ireland; 87 Centre for Molecular Medicine, University Medical Center Utrecht, Heidelberglaan 100, 3584 CX Utrecht, Netherlands; 88 Centre for Advanced Imaging, University of Queensland, Brisbane, Queensland Australia; 89 Program in Complex Trait Genomics, Institute for Molecular Bioscience, University of Queensland, St Lucia, Queensland Australia; 90 Department of Psychiatry, Amsterdam Public Health and Amsterdam Neuroscience, VU University Medical Center/GGZ inGeest, Amsterdam, The Netherlands, Oldenaller 1, 1081 HJ Amsterdam, The Netherlands; 91 CHU Sainte-Justine Research Center, Université de Montréal, Montréal, QC Canada; 92 Departments of Psychosis Studies, Institute of Psychiatry, Psychology & Neuroscience, King’s College London, London, United Kingdom; 93 Center for Neurobehavioral Genetics, University of California, Los Angeles, California, 90095 USA; 94 Rotman Research Institute, University of Toronto, Toronto, M6A 2E1 Canada; 95 Department of Psychiatry, University of Toronto, Toronto, M5S 1A1 Canada; 96 Center for Developing Brain, Child Mind Institute, New York, NY 10022 USA; 97 Department of Psychology, University of Toronto, Toronto, M5S 1A1 Canada; 98 Department of Psychiatry, Amsterdam Public Health and Amsterdam Neuroscience, VU University Medical, Amsterdam, Netherlands; 99 Departments of Radiology & Clinical Neuroscience, University of Calgary, Calgary, T2N 1N4 Canada; 100 Bioinformatics Service, Nucleus, University of Salamanca (USAL), 37007 Salamanca, Spain; 101 Department of Integrative Medical Biology, Linnéus väg 9, 901 87 Umeå, Sweden; 102 Centre for Population Neuroscience and Stratified Medicine, Social, Genetic and Development Psychiatry Centre, Institute of Psychiatry, Psychology and Neuroscience, King’s College London, 16 De Crespigny Park, SE5 8AF London, UK; 103 Psychiatry Imaging Group, MRC London Institute of Medical Sciences, Faculty of Medicine, Imperial College London, Hammersmith Hospital, Du Cane Road, W12 0NN London, UK; 104 Center for Integrative Genomics, University of Lausanne, Genopode building, CH-1015 Lausanne, Switzerland; 105 Department of Neurology, University Hospital of Trondheim, Edvard Griegs gate 8, N-7006 Trondheim, Norway; 106 Orygen, The National Centre of Excellence in Youth Mental Health, 35 Poplar Road, 3502 Parkville, New Mexico Australia; 107 Centre for Youth Mental Health, The University of Melbourne, 35 Poplar Road, 3502 Parkville, Victoria Australia; 108 Department of Psychiatry, VU University Medical Center, 1007 MB Amsterdam, The Netherlands; 109 Neuroscience Research Australia, Randwick, Australia; 110 School of Medical Sciences, University of New South Wales, Sydney, Australia; 111 Department of Clinical and Experimental Epilepsy, UCL Institute of Neurology, London, UK; 112 Chalfont Centre for Epilepsy, London, UK; 113 Dept of Psychiatry, University of Cape Town, Groote Schuur Hospital, Anzio Rd, 7925 Cape Town, South Africa; 114 MRC Unit on Risk & Resilience in Mental Disorders, Stellenbosch, South Africa; 115 Queensland Brain Institute, University of Queensland, St Lucia, Queensland Australia; 116 Institute for Community Medicine, University Medicine Greifswald, Greifswald, Germany; 117 Neuroimaging Unit, Technological Facilities. Valdecilla Biomedical Research Institute IDIVAL, Santander, Cantabria 39011 Spain; 118 Department of Psychology, Georgia State University, Atlanta, GA USA; 119 Department of Psychiatry, Stellenbosch University, TBH Francie van Zijl Avenue, 7500 Cape Town, South Africa; 120 Department of Psychiatry, 1 South Prospect Street, 5401 Burlington, Vermont USA; 121 Faculty of Electrical and Computer Engineering, University of Iceland, Reykjavik, Iceland; 122 MRC Social, Genetic and Developmental Psychiatry Centre, Institute of Psychiatry, Psychology and Neuroscience, King’s College London, 16 De Crespigny Park, SE5 8AF London, UK; 123 Faculty of Medicine, University of Iceland, Reykjavik, Iceland; 124 German Center for Neurodegenerative Diseases (DZNE), Rostock, Greifswald, Greifswald, Germany; 125 Centre for Advanced Imaging, University of Queensland, St Lucia, Queensland Australia; 126 Department of Psychiatry, Osaka University Graduate School of Medicine, Suita, Osaka Japan; 127 Department of Pediatrics, University of Montreal, Montreal, H3C 3J7 Canada; 128 Department of Medical Genetics, Oslo University Hospital, Kirkeveien 166, 424 Oslo, Norway

**Keywords:** Psychiatric disorders, Neuroscience

## Abstract

Carriers of large recurrent copy number variants (CNVs) have a higher risk of developing neurodevelopmental disorders. The 16p11.2 distal CNV predisposes carriers to e.g., autism spectrum disorder and schizophrenia. We compared subcortical brain volumes of 12 16p11.2 distal deletion and 12 duplication carriers to 6882 non-carriers from the large-scale brain Magnetic Resonance Imaging collaboration, ENIGMA-CNV. After stringent CNV calling procedures, and standardized FreeSurfer image analysis, we found negative dose-response associations with copy number on intracranial volume and on regional caudate, pallidum and putamen volumes (*β* = −0.71 to −1.37; *P* < 0.0005). In an independent sample, consistent results were obtained, with significant effects in the pallidum (*β* = −0.95, *P* = 0.0042). The two data sets combined showed significant negative dose-response for the accumbens, caudate, pallidum, putamen and ICV (*P* = 0.0032, 8.9 × 10^−6^, 1.7 × 10^−^^9^, 3.5 × 10^−12^ and 1.0 × 10^−4^, respectively). Full scale IQ was lower in both deletion and duplication carriers compared to non-carriers. This is the first brain MRI study of the impact of the 16p11.2 distal CNV, and we demonstrate a specific effect on subcortical brain structures, suggesting a neuropathological pattern underlying the neurodevelopmental syndromes.

## Introduction

Carriers of large recurrent copy number variants (CNVs) are at increased risk for developing autism spectrum disorders (ASD), schizophrenia or intellectual disability [1]. While the same CNV may confer risk for each of these neurodevelopmental disorders, carriers show remarkable phenotypic variability [2–5]. Magnetic resonance imaging (MRI) can help unravel possible underlying brain consequences associated with carrying these CNVs and provide novel insight into neuropathological mechanisms [2, 6–10].

**Fig. 1:**
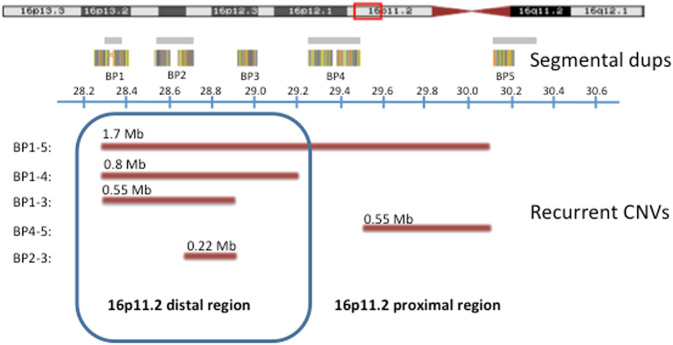
Recurrent CNVs in the 16p11.2 region. CNVs are indicated with reddish lines. All coordinates (in MB) are from the human genome build hg18/NCBI 36. This study includes CNVs overlapping the core 16p11.2 distal region (BP2–BP3) of 220 kb (blue box). These CNVs include the 16p11.2 distal BP2–BP3 (~220 kb), the 16p11.2 distal BP1–BP3 (~550 kb), the 16p11.2 distal BP1–BP4 (~800 kb) and the 16p11.2 distal-proximal BP1–BP5 (~1.7 MB) CNVs

The neural correlates of a recurrent CNV at the distal 16p11.2 locus have remained unexplored, despite being potentially very informative. Low copy repeats at the 16p11.2 locus drive the formation of recurrent CNVs (Fig. 1) [5, 11–15] whose carriers experience increased risk for various neurodevelopmental disorders [3, 16–25] or somatic traits and diseases [17, 26–30]. Within the 16p11.2 region, the segment with breakpoints (BP) at 28.3 and 28.9 Mb (hg18, BP1–BP3) is referred to as the distal region. Within this region, there is a minimal core segment from 28.7 to 28.9 Mb (hg18, BP2–BP3) (Fig. 1) that contains nine genes. The deletion is associated with obesity [26, 27], intellectual disability [26] and schizophrenia [20, 22] and the duplication has been associated with lower body mass index (BMI) [17, 31]. Both 16p11.2 distal CNVs are associated with autism spectrum disorder [17] and have been found in individuals with epilepsy [23]. Several studies have been published about the microstructural effect on the brain [6, 8, 32] and cognition [30, 33, 34] of the 16p11.2 proximal CNV (29.5–30.1 MB) (Fig. 1). In contrast, the biological basis of the 16p11.2 distal phenotypes, including a possible effect on brain structure and cognition remains unknown.

Large effect-size CNVs conferring risk for neurodevelopmental disorders including major psychiatric disorders [35] are rare (<0.25% in frequency). Assembling sufficiently powered MRI samples to detect effects of rare CNVs on brain morphometry is challenging. For instance, in the Icelandic population [2] and the UK Biobank [36], the frequencies of the 16p11.2 distal deletion are 0.019% and 0.012%, respectively. Likewise, the reciprocal duplication is found at a frequency of 0.038% and 0.030%, respectively. Hence, studying rare pathogenic CNVs like 16p11.2 distal calls for collaborative efforts. The ENIGMA-CNV consortium has collected a sample that currently includes 16,046 subjects with CNV and brain MRI data.

In recent studies, ‘mirrored phenotypes’ were described in 16p11.2 distal CNV allele carriers for both weight [17, 31] and head circumference [17]. On average, deletion carriers had increased BMI and head circumference, whereas duplication carriers had lower weight and smaller head circumference. Here we investigated gene dose response effects (i.e., effects dependent on the number of genomic copies at the 16p11.2 distal locus) on brain structural measures, including subcortical brain volumes, total surface area, mean cortical thickness and intracranial volume (ICV) in *n* = 6906 participants from primarily non-familial population samples, in addition to clinical cohorts, to resolve CNV effects relative to a general population average.

## Material and methods

### Discovery sample description

Supplementary Table 1 contains information on study design, participants, genotyping array, PFB-file and reference for previous description on individual inclusion and exclusion parameters for all 34 world-wide data sets in ENIGMA-CNV; altogether 16,046 individuals with CNV and MRI imaging data from the ENIGMA consortium. The 16p11.2 distal sample consisted of a subset of these individuals with twelve deletion carriers, twelve duplication carriers and 6882 non-carriers from eleven different cohorts and 14 scanner sites collected up until 1 July 2017. More demographic details are supplied in Supplementary Note 1 and Supplementary Table 2 (on CNV carriers).

### CNV calls and validation

See Supplementary note 2 for details on CNV calling and quality control. In short, carriers in the 16p11.2 European consortium cohort were identified based on report from the cytogeneticist. All other cohorts had CNVs called in a unified manner using PennCNV [37]. Appropriate population frequency (PFB)-files (Human Genome Build NCBI36/hg18) and GC (content)-model files for each data set were selected from the PennCNV homepage (Supplementary Table 1).

Samples were filtered based on standardized quality control metrics and CNVs overlapping the region of interest (16p11.2 distal BP2–BP3 and BP1–BP3) were identified and visualized with the R package iPsychCNV. The minimally affected 16p11.2 distal region was covered well by all the arrays in the study (Supplementary Figure 1). No carriers carried other genomic imbalances (as defined by Supplementary Table 3) except six 16p11.2 distal-proximal CNV (BP1–BP5) carriers from the 16p11.2 European consortium sample (Supplementary Table 2).

### Image acquisition and processing

Supplementary Table 4 outlines-specific technical details concerning scanners and acquisition parameters. The brain measures examined were obtained from structural MRI data collected at participating sites and processed locally following the ENIGMA protocol. The analysis was based on standardized image analysis, FreeSurfer, quality assurance and statistical methods as per the harmonized neuroimaging protocols developed within ENIGMA2 [38] and ENIGMA3 (http://enigma.ini.usc.edu/protocols/imaging-protocols). More details are supplied in Supplementary Note 3.

### Statistical analysis

Imaging data processing and CNV calling were performed locally whereupon downstream analysis was performed centrally in a mega-analysis with de-identified data.

The primary analysis for this paper focused on the full set of subjects including family members and data sets with patients to maximize the power to detect effects. Only one of a pair of duplicates was kept. Individuals with a minimum overlap of 0.4 to regions (R package iPsychCNV) with known pathogenic CNVs (Supplementary Table 3) were excluded from the analysis regardless of copy number status. Only scanner sites with individuals carrying a 16p11.2 distal deletion or duplication were included. See Supplementary Note 4 for description of control analyses excluding either (a) individuals with an established neurodevelopmental diagnosis, (b) children below age 18, (c) first and second-degree relatives or (d) carriers of the 1.7 MB 16p11.2 distal-proximal (BP1–BP5) CNV or (e) matching each carrier with four controls or (f) testing the effect of ancestry.

Brain measures were normalized in R 3.2.3 by an inverse normal transformation of the residual of a linear regression on the phenotype correcting for covariates. The final covariance-corrected values (covariates = age, age squared, sex, scanner site and ICV) were used in downstream analysis and are reported for each measure. ICV was not included as a covariate in the analysis of ICV. For analytic purposes, total cortical surface area and total average thickness were normalized in the same way as subcortical volumes. We also performed analysis excluding ICV from the covariates.

For the dose response analysis (i.e., the effect on brain structure of 16p11.2 distal copy number variation), a linear regression on the copy number state of the individuals (deletion = 1, normal = 2, duplication = 3) was performed using the following model: covariance-corrected brain measure ~ copy number (deletion = 1, non-carrier = 2, duplication = 3).

For comparison between groups, a two sample two-sided *t*-test assuming equal variance in all carrier/non-carrier groups was employed (R 3.3.2) where deletion or duplication carriers were compared either to each other or to non-carriers. Results were considered statistically significant if they exceeded a Bonferroni-corrected *P*-value (*P* = 0.05/10 regions = 0.005). We report the uncorrected *p*-values throughout the manuscript.

Effect size is calculated as the absolute effect size (the difference in mean between the two copy number groups in the *t*-test—which, in this case, equals Cohen’s *d* as the standard deviation of the normalized brain measures is one) and the estimate of beta in the linear regression. Plots were generated using R library ggplot2 v2.2.1 [39].

### deCODE replication sample

An independent sample of three 16p11.2 distal deletion and, six duplication carriers, as well as 832 non-carriers was obtained from deCODE Genetics, Iceland. CNVs were called with PennCNV as described previously and visually inspected. All 16p11.2 distal carriers were of the minimal 16p11.2 distal (BP2–BP3) CNV type. The individuals were scanned at one scanner site as previously described [7]. The statistical analysis was performed as for the primary discovery sample.

### Meta-analysis

A fixed effects model was used to generate summary effect size estimates using a restricted maximum likelihood estimator in the R-package metafor-package [40] (version 1.9-9) using the effect size and calculated SD (for comparison between groups) or standard error (for dose response) from the discovery and replication sample as input. More details can be found in Supplementary Note 5.

### IQ, BMI and gene expression analysis

Individuals aged 18–65 years were recruited for cognitive phenotyping based on a large genotyped sample from deCODE. The Icelandic version of the Wechsler Abbreviated Scale of Intelligence (WASIIS) [41, 42] was administered to 1693 non-carriers and all CNV carriers except one deletion carrier. Another 455 controls and one deletion carrier were tested with two subtests, Vocabulary and Matrix Reasoning, from the Wechsler Adult Intelligence Scale (WAIS-III) [43]. More details on tests are available in Supplementary Note 6. Carriers of known pathogenic CNVs (Supplementary Table 3) besides 16p11.2 distal as well as individuals with neurodevelopmental or psychiatric diagnoses were excluded from the analysis. IQ data were not normally distributed and, consequently, the non-parametric Kruskal–Wallis test (R, v3.2.3) was used to test differences in IQ between carrier groups. To test pairwise differences (deletion carriers versus non-carrier-controls, duplication carriers versus non-carrier-controls, deletion versus duplication carriers), we used Wilcoxon rank test in R. We applied a significance threshold of 0.05, without correction for multiple testing since this was secondary analyses. For description of BMI and gene expression analysis, see Supplementary Note 7.

## Results

### Study participants

In the ENIGMA-CNV discovery data set, we identified 12 16p11.2 distal deletion carriers and 12 duplication carriers scanned at 14 MRI scanners, and 6882 non-carriers investigated at the same MRI scanners. Demographic data are shown in Table 1. Most CNV carriers exhibited the minimal 16p11.2 distal CNV type (BP2–BP3) (Fig. 1), four CNVs were of the extended type (BP1–BP3) and six CNVs extended into the 16p11.2 proximal region (BP1–BP5) (Supplementary Table 2, Supplementary Figure 1). None of the participants carried additional known pathogenic CNVs (Supplementary Table 3).

**Table 1:** Demographic data, discovery and replication data

	Discovery, ENIGMA-CNV				Replication, deCODE			
	del	con	dup	*P*	del	con	dup	*P*
Individuals	12	6882	12		3	832	6	
Age (mean (sd))	27.8 (20.4)	43.5 (20.0)	31.2 (19.6)	0.003	48.7 (14)	46.2 (12)	47.0 (15)	NS
Sex = female (%)	6 (50%)	3724 (54%)	3 (25%)	NS	2 (67%)	502 (60%)	1 (17%)	NS
Established diagnosis, *n* (%)	7 (58%)	336 (4.9%)	3 (25%)	<0.001	0 (0%)	158 (19%)	2 (33%)	NS
*Diagnosis type (%)*^a^								
ADHD	1 (8.3%)^b^	2 (<0.1%)				149 (18%)	2 (33%)	
Autism			2 (17%)			1 (0.1%)		
Bipolar	1 (8.3 %)	100 (1.5 %)				7 (0.8%)		
Dysthymia	1 (8.3 %)^c^	1 (<0.1%)						
Epilepsy	1 (8.3%)	101 (1.5%)						
Enuresis	1 (8.3%)							
Language disorder	1 (8.3%)^c^							
MDD		13 (0.2%)	1 (8.3%)^d^					
Schizophrenia	1 (8.3%)	119 (1.7%)				1 (0.1%)		
Scanner sites	7	14	9		1	1	1	
Types of arrays^e^	8	11	9		Various	Various	Various	
Children, *n* (%)	5 (41.7%)	788 (11.5%)	5 (41.7%)	<0.001	0 (0)	0 (0)	0 (0)	
Close relative in data set, *n* (%)	5 (41.7%)	1994 (30.8%)	2 (16.7%)	NS	3 (0%)	832 (0%)	6 (0%)	

Age, sex, established diagnosis, diagnosis type, number of scanner sites, types of arrays, children (number of individuals below 18 years) and individuals with close relatives (>3^rd^ degree relatives) in the data set. *P* (*p*-value) is based on a *χ*^2^-test for categorical values and ANOVA for continuous values (R-package, TableOne)

*ADHD* attention deficit disorder, *MDD* major depressive disorder, *SCZ* schizophrenia, *del* deletion carrier, *con* non-carriers, *dup* duplication carrier, *NS* non-significant

^a^Diagnosis type specifies the total number within each carrier group with an established neurodevelopmental or psychiatric diagnosis

^b^In addition to Specific Learning disorder

^c^In addition to Moderate ID and Speech Sound disorder

^d^In addition to Social Anxiety disorder

^e^The arrays for ENIGMA-CNV are specified in Sup Table 1

Of 24 CNV carriers, 10 had an established neurodevelopmental diagnosis (Supplementary Table 2). The remaining carriers either did not have one or were recruited in studies from which diagnostic information was not available (Supplementary Table 2, Table 1).

There was a significant age difference between the groups (ANOVA, *P* = 0.003); the non-carriers were older (mean age 43.5 years) in comparison to the deletion (27.8 years) and duplication carriers (31.2 years). In addition, an established neurodevelopmental diagnosis was found in a significantly smaller proportion of non-carriers (4.9%) in comparison to deletion (58%) and duplication carriers (25%) (Table 1).

### Brain imaging results in the discovery sample

**Fig. 2:**
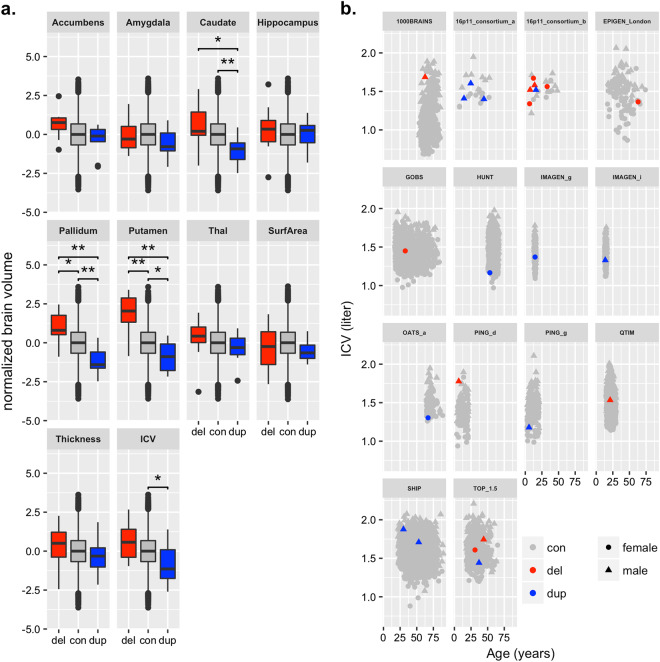
Measures of caudate, pallidum, putamen and ICV show a dose response to differences in copy number in the 16p11.2 distal region. All analyses were corrected for age, age squared, sex, scanner site and ICV (except for ICV). Deletion carriers (del) in red, non-carriers (con) in grey and duplication carriers (dup) in blue, respectively. **a** Boxplots of subcortical volumes, surface area and thickness and ICV. The normalized brain values are presented. Boxplots represent the mean. Significant differences after Bonferroni correction between groups are noted as *P < 0.005, **P < 0.0005. Centre line represents median, box limits are the upper and lower 25% quartiles, whiskers the 1.5 interquartile range and the points are the outliers. **b** Bivariate plot of age versus uncorrected ICV

After correction for age, age squared, sex and scanner site, we found a significant negative correlation between the number of 16p11.2 distal copies (deletion = 1, non-carrier = 2, duplication = 3) and ICV (*β* = −0.71, *P* = 5.1 × 10^−4^) (Table 2, Fig. 2a) after correction for multiple testing (significance threshold *P* < 0.005 = 0.05/10 brain structures analysed), showing smaller ICV in duplication carriers compared to deletion carriers. The uncorrected ICV plotted against age stratified by scanner site are shown in Fig. 2b.

**Table 2:** Dose response of 16p11.2 distal copy number on subcortical volumes

	ENIGMA-CNV, discovery		deCODE, replication		Combined (discovery + replication)						
Brain measure	*β*	*P*	*β*	*P*	*Q*	*p*(*Q*)	I2	*β*	CI, *β*, lower	CI, *β*, upper	*P*
Accumbens	−0.49	0.025	−0.65	0.05	0.163	0.69	0	−0.54	−0.9	−0.18	0.0032*
Amygdala	−0.21	0.31	−0.13	0.7	0.043	0.84	0	−0.19	−0.52	0.15	0.27
Caudate	−0.87	2.0E–05**	−0.46	0.17	1.13	0.29	11.4	−0.76	−1.1	−0.42	8.9E–06**
Hippocampus	−0.14	0.48	−0.03	0.94	0.0813	0.78	0	−0.11	−0.45	0.22	0.52
Pallidum	−1.06	2.2E–07**	−0.95	0.0042*	0.0813	0.78	0	−1.03	−1.37	−0.7	1.7E–09**
Putamen	−1.37	1.8E–11**	−0.7	0.034	3.01	0.083	66.8	−1.19	−1.53	−0.85	3.5E–12**
Thalamus	−0.33	0.11	−0.37	0.27	0.0105	0.92	0	−0.34	−0.69	0.01	0.054
Surface Area	−0.09	0.66	0.14	0.68	0.355	0.55	0	−0.03	−0.36	0.31	0.87
Thickness	−0.29	0.16	−0.02	0.94	0.49	0.48	0	−0.22	−0.55	0.12	0.2
ICV	−0.71	5.1E–04*	−0.54	0.1	0.194	0.66	0	−0.66	−1	−0.33	1.0E–04**

The effect size (β of the linear regression) is presented. A linear regression based on the copy number state of the individuals (deletion = 1, non-carrier = 2, duplication = 3) was performed on normalized brain measures correcting for age [2], age, sex and scannersite (and ICV) in the ENIGMA-CNV (discovery) and deCODE (replication) cohorts. Results were considered statistically significant if they were below a Bonferroni-corrected *P*-value of 0.005 (0.05/10 regions). A final effect size estimate of the combined sample was obtained using a fixed effects meta-analysis framework

*CI* confidence interval, *Q* statistics for the test for heterogeneity, *p(Q)* *p*-value for the test for heterogeneity, *I2* heterogeneity levels

*P < 0.005

**P < 0.0005

We evaluated whether the 16p11.2 distal CNV affected seven subcortical (accumbens, caudate, putamen, pallidum, amygdala, hippocampus and thalamus) and two cortical (total surface area and mean cortical thickness) phenotypes. After adjusting subcortical and cortical volumes for age, age squared, sex, scanner site and ICV, the volumes of caudate, pallidum and putamen were negatively associated with the number of 16p11.2 distal copies with significance at the multiple testing threshold (*β* = −0.87, *P* = 2.0 × 10^−5^; *β* = −1.06, *P* = 2.2 × 10^−7^ and *β* = −1.37, *P* = 1.8 × 10^−11^, respectively) (Table 2, Fig. 2a). Plotting the unadjusted volumes of caudate, pallidum and putamen against the age of participants revealed a consistent pattern (deletion carriers with larger and duplication carriers with smaller subcortical volumes in comparison to non-carriers) for all scanner sites for putamen and pallidum volume and almost at all sites for caudate (Supplementary Figure 3a-c). This shows that our findings are robust and not dependent upon a few scanner sites.

To assess non-specific associations, we re-analyzed subcortical volumes without correcting for ICV. As expected, the absolute effect sizes of copy number on the volumes of caudate, pallidum and putamen increased (*β* = −1.18, *P* = 6.8 × 10^−9^; *β* = −1.27, *P* = 4.7 × 10^−10^ and *β* = −1.57, *P* = 1.4 × 10^−14^, respectively) and the association with the volumes of the rest of subcortical structures, except amygdala and hippocampus, became significant (Supplementary Table 5).

To test for the presence of nonlinear differences between deletion and duplication carriers, and deletion or duplication carriers and non-carriers, we conducted individual *t*-tests between these groups. We confirmed a negative dose response with increasing copy number for the volumes of the caudate, pallidum, putamen and for ICV but no additional structures revealed significant associations at significance threshold of <0.005 (Table 3, Supplementary Table 6, Supplementary Figure 3a-c).

**Table 3:** *T*-test on subcortical volumes between different 16p11.2 distal copy number groups

	ENIGMA-CNV, discovery		deCODE, replication		Combined, discovery + replication								
Brain measure	Cohens *D*	*P*	Cohens *D*	*P*	*n*, CN1	*n*, CN2	*Q*	*p*(*Q*)	I2	Cohens *D*	CI, lower	CI, upper	*P*
*Deletion vs duplication*													
Accumbens	−0.99	0.023	−1.86	0.044	13	17	0.99	0.32	0	−1.17	−1.87	−0.47	1.0E–03*
Amygdala	−0.42	0.32	−0.27	0.73	15	18	0.031	0.86	0	−0.38	−1.09	0.32	0.28
Caudate	−1.74	0.0013*	−1.25	0.25	15	18	0.251	0.62	0	−1.63	−2.44	−0.82	8.0E–05**
Hippocampus	−0.29	0.57	−0.16	0.79	15	18	0.034	0.85	0	−0.22	−0.91	0.47	0.53
Pallidum	−2.11	1.8E–05**	−2.19	0.0038*	15	18	0.0091	0.92	0	−2.13	−2.79	−1.47	3.2E–10**
Putamen	−2.73	5.3E–06**	−1.67	0.089	15	18	0.77	0.38	0	−2.58	−3.41	−1.74	1.4E–09**
Thalamus	−0.66	0.18	−0.85	0.045	15	17	0.079	0.78	0	−0.76	−1.41	−0.1	0.024
Surface area	−0.18	0.69	−0.04	0.96	15	18	0.012	0.91	0	−0.16	−0.98	0.66	0.7
Thickness	−0.58	0.25	0.17	0.84	15	18	0.53	0.47	0	−0.41	−1.26	0.45	0.35
ICV	−1.41	0.0084	−0.70	0.4	15	18	0.65	0.42	0	−1.19	−1.99	−0.4	0.0034*
*Deletion vs non-carrier-controls*													
Accumbens	−0.54	0.088	−1.78	0.0019*	13	2467	3.1	0.078	67.8	−0.73	−1.22	−0.23	0.0039*
Amygdala	0.08	0.79	−0.15	0.8	15	3578	0.11	0.74	0	0.04	−0.49	0.56	0.89
Caudate	−0.63	0.027	−1.12	0.05	15	3574	0.46	0.5	0	−0.79	−1.45	−0.12	0.02
Hippocampus	−0.26	0.37	−0.23	0.69	15	3578	0.0031	0.96	0	−0.24	−0.75	0.27	0.35
Pallidum	−0.82	0.0043*	−1.52	0.0081	15	3577	0.87	0.35	0	−0.95	−1.51	−0.38	1.0E–03*
Putamen	−1.72	2.4E–09**	−1.24	0.031	15	3584	0.18	0.67	0	−1.67	−2.35	−0.99	1.5E–06**
Thalamus	−0.3	0.3	−0.59	0.3	15	3571	0.24	0.62	0	−0.42	−1	0.16	0.15
Surface area	0.24	0.4	−0.50	0.38	15	4312	0.41	0.52	0	0.14	−0.61	0.9	0.71
Thickness	−0.28	0.32	0.43	0.45	15	4312	0.66	0.42	0	−0.13	−0.83	0.58	0.73
ICV	−0.44	0.13	0.23	0.7	15	4314	0.97	0.33	0	−0.26	−0.85	0.32	0.38
*Non-carrier-controls vs duplication*													
Accumbens	−0.34	0.26	−0.08	0.84	4433	17	0.25	0.62	0	−0.27	−0.73	0.19	0.26
Amygdala	−0.46	0.11	−0.13	0.76	4533	18	0.41	0.52	0	−0.36	−0.83	0.11	0.13
Caudate	−1.04	3.0E−04**	−0.12	0.77	4617	18	1.9	0.17	46.2	−0.92	−1.37	−0.47	6.1E−05**
Hippocampus	0.06	0.85	0.08	0.84	4557	18	0.0030	0.96	0	0.07	−0.35	0.49	0.75
Pallidum	−1.1	0.00013**	−0.66	0.1	4504	18	2.3	0.13	56.5	−0.86	−1.14	−0.58	1.9E−09**
Putamen	−0.85	0.0031*	−0.44	0.28	4454	18	0.99	0.32	0	−0.7	−1.09	−0.31	4.9E−04**
Thalamus	−0.33	0.27	−0.25	0.54	4562	17	0.063	0.8	0	−0.27	−0.5	−0.03	0.025
Surface area	−0.33	0.25	0.46	0.26	4575	18	3.0	0.086	66.1	−0.19	−0.53	0.16	0.28
Thickness	−0.28	0.33	−0.24	0.55	4578	18	0.0054	0.94	0	−0.27	−0.75	0.21	0.28
ICV	−0.88	0.0023*	−0.93	0.022	4690	18	0.0068	0.93	0	−0.9	−1.45	−0.35	0.0014*

Deletions versus duplication carriers, deletion carriers versus non-carriers and non-carriers versus duplication carriers in ENIGMA (discovery), deCODE (replication) and the combined sample. *T*-tests were performed on normalized values of brain measures correcting for age [2], age, sex and scanner site (and ICV). Results were considered significant if they were below a Bonferroni-corrected *P*-value of 0.005 (0.05/10 regions). A final Cohen’s *d*-effect size estimate of the combined sample was obtained using a fixed effects meta-analysis framework

*CI* confidence interval, *Q* statistics for the test for heterogeneity, *p(Q)* *p*-value for the test for heterogeneity, *I2* heterogeneity levels

*P < 0.005

**P < 0.0005

To confirm the validity of the results, we carefully checked for the impact of removing subjects known to carry a neurodevelopmental diagnosis, children below age 18, first and second-degree relatives or CNV carriers whose CNVs extended into the 16p11.2 proximal region (Supplementary Tables 5 and 6). Likewise, we redid the analysis matching each carrier with four non-carriers for sex, age, diagnosis status (with/without neurodevelopmental diagnosis) and scanner site and finally in a separate analysis we controlled for population stratification in cohorts with available ancestry (Supplementary Table 7). None of these analyses changed the main results.

### Replication in an independent cohort

**Fig. 3:**
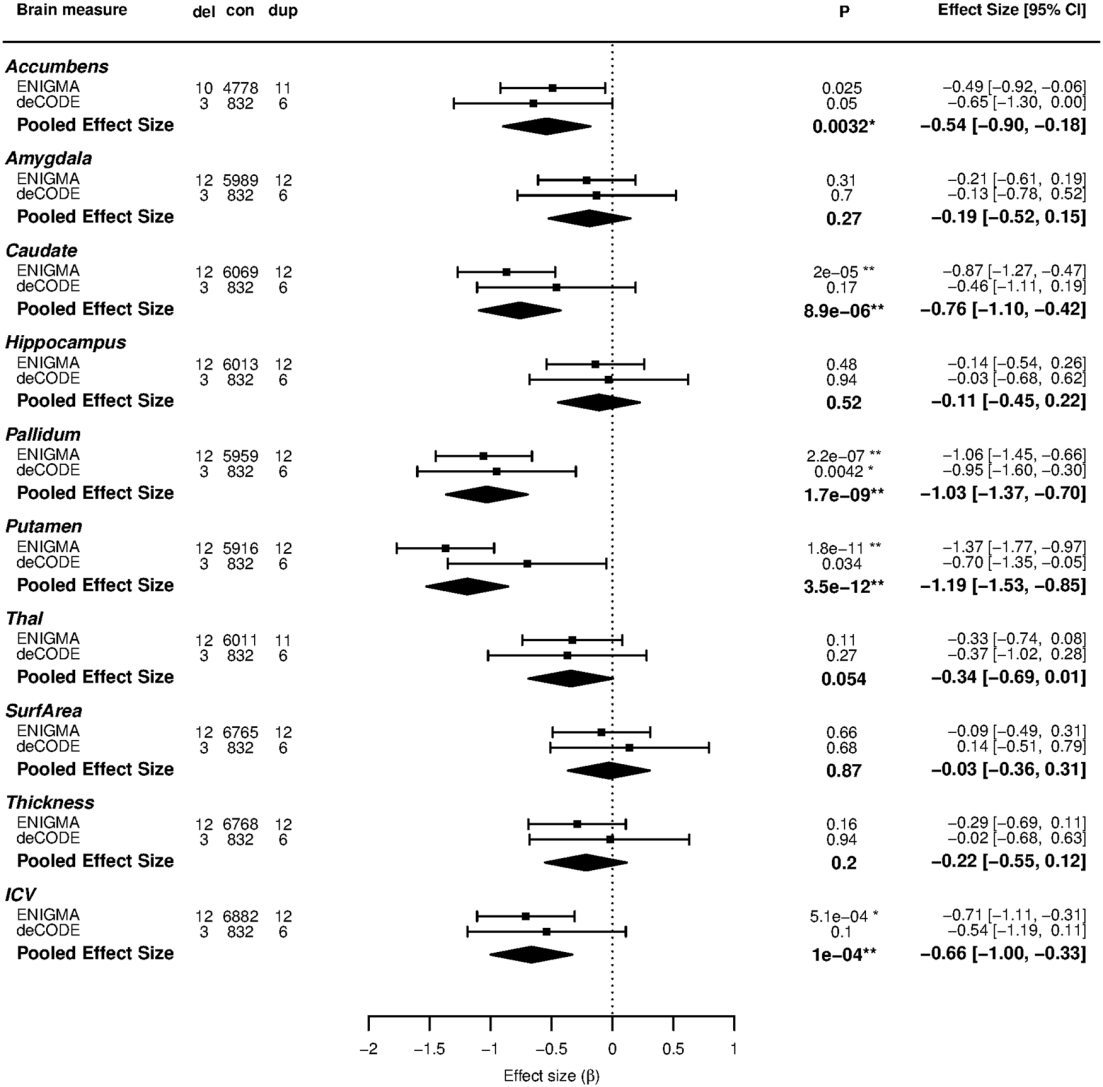
Forest plots on the dose response of copy number on subcortical volumes, surface area, thickness and ICV. The effect size (β of the linear regression) at each site for each measure is shown by the position on the x-axis. Standard error is shown by the horizontal line. A summary polygon shows the results when fitting a random-effects model to the two groups: ENIGMA-CNV discovery and deCODE replication samples. del, con and dup denote the number of individuals in each analysis. *P < 0.005, **P < 0.0005. Effect size and confidence intervals are to the right

We performed replication of the subcortical findings in an Icelandic MRI sample (deCODE) comprising 841 individuals (3 deletion and 6 duplication carriers, 832 non-carriers) (Table 1). The negative correlation between the number of 16p11.2 distal copies and the volume of pallidum was confirmed (*β* = −0.95, *P* = 0.0042) (Fig. 3, Table 2) at a significance threshold of <0.005. For volumes of the caudate, putamen and for ICV, effects were in the same direction as in the discovery sample, albeit not significant (*β* = −0.46, *P* = 0.17; *β* = -0.70, *P* = 0.034; *β* = −0.54, *P* = 0.10, respectively) (Fig. 3, Table 2). Apart from cortical surface area, we observed the same direction of effect in the replication sample as in the discovery sample (Table 2, Fig. 3). For nonlinear differences, all directions of effect were the same for subcortical volumes in the discovery and replication data sets (Table 3, Supplementary Figure 4a-c).

The combined analysis of the ENIGMA and the deCODE samples is shown in Table 2 and Fig. 3. Volumes of caudate, pallidum, putamen and ICV decreased with increasing number of 16p11.2 distal copies (*β* = −0.76, *P* = 8.9 × 10^−6^; *β* = −1,03, *P* = 1.7 × 10^−9^; *β* = −1.19, *P* = 3.5 × 10^−12^; *β* = −0.66, *P* = 1.0 × 10^−4^, respectively). In the combined analysis, the volume of the accumbens also revealed a significant association with the 16p11.2 distal copy number (*β* = −0.54, *P* = 0.0032) (Tables 2 and 3, Fig. 3, Supplementary Figure 4a-c).

### Intelligence quotient (IQ)

Full scale IQ data were available for four 16p11.2 distal deletion and twelve duplication carriers and 2148 non-carriers from the Icelandic sample. None of these individuals had an established neurodevelopmental diagnosis, or other known pathogenic CNVs (as defined by Supplementary Table 3). Analysis showed a significant difference in IQ between groups (*P* = 0.0042). Both deletion (median IQ = 68.5) and duplication carriers (median IQ = 93) presented a significantly lower IQ (*P* = 0.011, *P* = 0.035) than non-carriers (median IQ = 101.5) (Supplementary Table 9, Supplementary Figure 5) at a significance threshold of *P* < 0.05.

### Body mass index (BMI)

BMI data for mega-analysis were available for six cohorts from ENIGMA-CNV counting seven deletion and seven duplication carriers in addition to 1880 individuals without a 16p11.2 distal CNV (Supplementary Table 9). BMI *z*-scores were different between the carrier groups (Kruskal–Wallis, *P* = 0.009, Supplementary Figure 6, Supplementary Table 10). Duplication carriers had significantly lower BMI *z*-scores (median; SD = −0.65; 1.61) than the non-carriers (0.43; 1.19; *P* = 0.048). Also, the duplication carriers tended to have lower BMI *z*-scores than the deletions (−1.56; 1.1, *P* = 0.052; Supplementary Figure 6, Supplementary Table 10) and deletion carriers tended to have higher BMI *z*-scores than non-carriers (*P* = 0.18) (Supplementary Table 10). For detailed information on individual carriers, see Supplementary Table 2.

## Discussion

This is the first study to determine the brain structure underpinnings of the 16p11.2 distal recurrent CNVs. We found a common denominator for 16p11.2 distal carriers across different clinical phenotypes in a dose response effect of copy number on the volumes of basal ganglia (caudate, putamen and pallidum) (Table 2, Figs. 2 and 3). The observed associations were independent of the presence of neurodevelopmental diagnosis and the ancestry of participants (Supplementary Tables 5 and 7). These effects were consistent in the independent replication sample (Fig. 3, Tables 2 and 3). Together with the result of lower IQ in carriers, these findings provide new insight into genetic mechanisms of brain structures and pathobiological processes involved in neurodevelopmental disorders.

There are nine genes in the core 16p11.2 distal (BP2–BP3) region. We tested the expression level of these in blood in available transcript data from two of our deletion carriers (BP1–BP3) and compared with 234 non-carriers (Supplementary Figure 7, Supplementary Table 11). Several transcripts of the nine genes were relatively decreased in blood. Due to the low numbers, only trends can be suggested from these data but we find the down-regulation of *LAT* gene expression to ~65% in 16p11.2 distal carriers (Supplementary Figure 7, Supplementary Table 11) particular interesting due to recent results in zebrafish: These showed that of the nine genes in 16p11.2 distal, only over-expression of *LAT* induced a decrease in cell proliferation in the brain with a concomitant microcephaly phenotype [44]. In parallel, *LAT* knockout mice showed brain anatomy changes [44]. According to the Allen Brain Atlas, *LAT* shows the highest expression in cerebellum and structures of the basal ganglia (data not shown). Thus, high expression of *LAT* overlaps with the position of brain structural changes identified in the present study. This further implicates *LAT*, an immune signaling adaptor, as a possible dosage-dependent driver of the CNV-associated brain phenotypes including the basal ganglia.

By comparing the effect of a range of CNVs on the brain, it is possible to identify patterns of effects related to the genes involved, and thus learn about biological mechanisms. All three CNVs previously shown to have an effect on ICV, 16p11.2 proximal [6, 8], 22q11 [9] and Williams Syndrome [45], have concomitantly identified an effect on either cortical surface area and/or cortical thickness. We observed no effect on cortical surface area or cortical thickness in 16p11.2 distal carriers (Table 2, Figs. 2 and 3). This does not rule out the presence of smaller effects on individual cortical areas. However, it may suggest a different impact on brain development mechanisms between these three CNVs [6, 8, 9, 45] and 16p11.2 distal.

CNVs in the two neighboring regions 16p11.2 distal and 16p11.2 proximal show overlapping phenotypes: they both dispose to various neurodevelopmental diseases and both show a positive dose response for head circumference and weight [17]. In addition, we found negative dose response effects for the 16p11.2 distal CNV on ICV, putamen and caudate volumes with effect size estimates comparable to those previously reported for the 16p11.2 proximal CNV [8]. Recently, a lymphoblastoid cell line study of chromosomal interaction in the 16p11.2 region suggested that the two adjacent 16p11.2 distal and 16p11.2 proximal regions (Fig. 1) interact [17]. The identified brain commonalities could further support a mechanism in which the similar phenotypic patterns are caused by disruption of the chromatin structure surrounding the entire 16p11.2 region [17, 32].

In this study, the CNVs (3 deletions and 3 duplications) of six carriers extend into the adjacent 16p11.2 proximal region. Redoing the analysis with exclusively 16p11.2 distal carriers did not result in a change in effect size (Supplementary Tables 5 + 6), suggesting that 1.7 MB distal-proximal (BP1–BP5) CNV carriers are not the main cause of the signal. A previous analysis from Loviglio et al. [44] suggests an additive effect of two 16p11.2 regions (distal + proximal) on human head circumference and weight. Together with our data, this indicates both separate and overlapping effects for the two CNVs and underlines the importance of studying specific CNVs independently despite overlapping phenotypes.

Interestingly, deletions and duplications of both 16p11.2 proximal and distal CNVs are associated with ASD [17, 19]. However, only the proximal duplication and the distal deletion are associated with schizophrenia [20, 22]. This difference in phenotype association between these bordering CNVs may indicate specific differences in the pathological mechanisms of ASD and schizophrenia.

We observed a decrease in absolute effect sizes for putamen and pallidum after removing individuals with a neurodevelopmental diagnosis (Supplementary Table 5). This is consistent with the enlargements of putamen and pallidum associated with duration of illness in schizophrenia [46], which may partly reflect the cumulative effect of antipsychotic medication on basal ganglia volumes [47]. The observed dose response effect on ICV is in agreement with previous findings on head circumference [17], as are the dose response effect on BMI (Supplementary Figure 6, Supplementary Table 10) [17, 31]. One of the strengths of this study is the inclusion of non-clinical samples allowing for estimates closer to the actual carrier population. Unfortunately, the small number of CNV cases does not provide enough power to investigate preferential alterations in deletion or duplication carriers.

To conclude, the present findings of negative dose-response effects of copy number on ICV and volumes of caudate, pallidum and putamen, with no effect on cortical measures, suggest a specific effect on basal ganglia structures of the 16p11.2 distal CNV. These results provide novel insight into genetic factors determining basal ganglia volumes and suggest specific pathobiological mechanisms involved in the development of neurodevelopmental disorders.

## Electronic supplementary material

Supplementary information

Supplementary figures

Supplementary tables
