# Supplementary material for: Dose response of the 16p11.2 distal copy number variant on intracranial volume and basal ganglia
Source: Mol Psychiatry. 2018 Oct 3;25(3):584–602. doi: 10.1038/s41380-018-0118-1 (PMC7042770; doi:10.1038/s41380-018-0118-1)
Supplement: Supplementary file 1 — Supplementary information [file 41380_2018_118_MOESM1_ESM.docx]

**Supplementary Information:**

**Dose response of the 16p11.2 distal copy number variant on intracranial volume and basal ganglia**

**Supplementary Note 1: Description of datasets and participants and test for differences in demographics**

**Supplementary Note 2: Details on quality control of CNVs.**

**Supplementary Note 3: Details on imaging analysis**

**Supplementary Note 4: Description of additional control analyses**

**Supplementary Note 5: Details on meta-analysis**

**Supplementary Note 6: Details on IQ measurements.**

**Supplementary Note 7: Description of BMI and gene expression analysis**

**Supplementary Note 1: Description of datasets and participants and test for differences in demographics**

A minority (4.9%) of the total 16p11.2 distal sample was affected with neurodevelopmental or psychiatric disorders (the major groups being epilepsy, schizophrenia, major depression, bipolar disorder or Attention Disorder Hyperactivity Disorder; ADHD) (Table 1). The ascertainments varied with two population-based twin studies (QTIM, OATS), one family study (GOBS), one epilepsy study (EPIGEN), one neurodevelopmental cohort (16p11.2 European consortium), five population based studies (HUNT, Imagen, 1000BRAINS, SHIP, PING) and one case-control study for psychiatric diseases (TOP) (Supplementary Table 1). Tests for differences between groups for demographic data applied a test included in the R package tableone v0.7.3 – chi square test with continuity correction for categorical values and ANOVA for continuous variables.

Family information was based on pi-hat estimated for pairs of individuals based on the genotyping array. For the 16p11.2 European consortium individuals, information on relatedness was based on self-report. For the analysis excluding family members, only one relative (if more than two) from pairs with pi-hat >0.2 was kept. All participants gave written informed consent and sites involved obtained ethical approvals.

**Supplementary Note 2: Details on quality control of CNVs.**

The genotypes used in the current study were obtained by genotyping with commercially available platforms, performed at participating sites for each cohort (Supplementary Table 1). In the case of cohorts primarily consisting of Asian and African individuals, a PFB-file was generated through PennCNV compile_pfb.pl and using all genotyping arrays from the cohort (Supplementary Table 1).

Only samples with standard deviation (SD) of normalized intensity (LRR) <0.35, B allele frequency (BAF) drifting value <0.01 and wave factor value between -0.05 and 0.05 were included. Adjacent CNVs separated by a gap less than 20% of the combined length of the two CNVs were merged until no more gaps of <20% existed, and CNVs based on less than 15 SNPs were excluded.

CNVs overlapping the region of interest (16p11.2 distal BP2-BP3 and BP1-BP3) were identified and visualized with the R package iPsychCNV SelectSamplesFromROI with parameters OverlapMin = 0 and OverlapMax = 3. A search was also performed for the 16p11.2 BP1-BP5 CNV (including both the 16p11.2 distal and 16p11.2 proximal CNVs) but none were identified. To exclude false-positives and include possible false negatives, LRR- and BAF-plots of the 16p11.2 distal region of all individuals in the analysis including non-carriers were generated with R package iPsychCNV StackPlot and visually inspected. No false positive or negative CNVs were identified.

Carriers in the 16p11.2 European consortium cohort were identified based on report from the cytogeneticist who did the genetic test in the clinic and was thus based on a variety of methods - MLPA (Multiplex Ligation-dependent Probe Amplification), CGH (comparative genomic hybridization) array, Agilent 400K CGH, FISH (Fluorescent In Situ Hybridization) - the identification method for each individual carrier is noted in Supplementary Table 2. Non-carriers in the 16p11.2 European consortium cohort were either selected from the general population (excluding individuals with a neurodevelopmental or psychiatric diagnosis) or familiar controls who tested negative for the 16p11.2 distal and proximal CNV - five of the latter had an established diagnosis of either MDD, ADHD or Persistent Depressive Disorder (Table 1).

**Supplementary Note 3: Details on imaging analysis**

Each site contributed volumes for the left and right hemispheres of the accumbens, caudate, putamen, pallidum, amygdala, hippocampus and thalamus in addition to right and left hemisphere total surface area and mean cortical thickness as well as estimated intracranial volume (ICV). The total volume of each subcortical structure, total surface area and thickness was calculated by adding the left and right.

**Supplementary Note 4: Description of additional control analyses**

Both for dose response analysis for comparison between groups analysis, we re-analysed the data excluding individuals with an established psychiatric or neurodevelopmental diagnosis (“NON-AFFECTED”) to verify that detected effects were not due to disease alone. Likewise, adults-only analyses excluding individuals below age 18 years were performed (“ADULTS”). An analysis was performed removing the carriers of the 1.7 MB 16p11.2 distal-proximal (BP1-BP5) carriers (“REMOVING 16p11.2 DISTAL-PROXIMAL CARRIERS”), thus leaving only carriers of the 16p11.2 distal CNV to rule out that these carriers were driving the signal. Finally, a matched controls analysis was carried out (“MATCHED CONTROLS”), in which the R package Matchit v2.4 was used to match each CNV carrier with four non-carriers based on gender, age, diagnosis status (with or without a known diagnosis) and scanner site. In the case of the 16p11.2 European consortium samples, most matched non-carriers did not carry a diagnosis and only 2-3 matched non-carriers were available for each carrier. Results are found in Supplementary Tables 5-6.

For the dose response analysis, any effect of population stratification was checked in a subset of samples from which MDS components, calculated based on standardized multidimensional scaling analyses of genome-wide genotype data conducted at each site, was available. Thus, in addition to the covariates in “plusICV” (age, age squared, sex, scanner site and ICV), we added four MDS-components (C1, C2, C3 and C4) into the model. The comparable analysis for the same individuals was done without correcting for MDS-components for comparison. Results are found in Supplementary Table 7.

All dose response and comparison between groups analysis were redone excluding first and second-degree relatives. Results are found in Supplementary Tables 5 and 6.

**Supplementary Note 5: Details on meta-analysis**

Heterogeneity levels (*I*^2^) were estimated for each structure, which gives the percent of the total variance in effect size that can be explained by heterogeneity alone. Lower values of I^2^ indicate lower variance in the effect size across studies. Values less than 25% are typically considered to represent low heterogeneity ^1^*.*

**Supplementary Note 6: Details on IQ measurements.**

The psychologists and psychiatrists evaluating all subjects were blind to genotype.  The Icelandic version of the Wechsler Abbreviated Scale of Intelligence (WASIIS)^2, 3^, which includes four subtests (Vocabulary, Similarities, Block Design and Matrix Reasoning), was administered to 1693 controls and all CNV carriers except one deletion carrier. Another 455 controls and one deletion carrier were tested with two subtests, Vocabulary and Matrix Reasoning, from the Wechsler Adult Intelligence Scale (WAIS-III)^4^. As Icelandic norms are unavailable for WAIS-III, these scores were combined with WASIIS scores using general cognitive ability (an extracted g factor) as an indicator of the comparability of the two control groups.

**Supplementary Note 7: Description of BMI and gene expression analysis**

**BMI:** BMI information for mega-analysis was available from six cohorts - GOBS, OATS, HUNT, TOP, 16p11.2 European consortium and Imagen. All individuals within the ENIGMA-CNV study with BMI information were included, regardless of CNV carrier status. Only one relative (if more than two) from pairs with pi-hat >0.2 was kept. Numbers for each carrier and cohort is presented in Supplementary Tables 2 and 9, respectively. Z-scores for BMI were estimated based on age- and sex-normed measurements from European and Swiss anthropometric normative data. The BMI data was not normally distributed and thus the effects of the genetic groups were analyzed with the non-parametric Kruskal-wallis test and post-hoc Wilcoxon rank test (R, v3.2.3) and were corrected with Bonferroni correction. Mean, SD, median, min and max for each respective cohort were calculated with basic R. BMI information was available for 1000BRAINS and SHIP cohorts, although not for mega-analysis. For these two cohorts, the BMI quantile for each carrier within their respective cohort was calculated and presented. Obesity is normally defined as BMI>30 kg/m^2^ and low weight as BMI<18 kg/m^2^ in adults.

**Gene Expression**: Blood samples from the TOP cohort were collected using Tempus Blood RNA Tubes (Life Technologies Corporation, Carlsbad, California, USA). Total RNA was extracted with ABI PRISM 6100 Nucleic Acid PrepStation and TEMPUS 12-port RNA Isolation Kit according to manufacturer's protocol.

From each sample, 200 ng of total RNA was biotin-labeled and amplified using the Illumina TotalPrep-96 RNA Amplification Kit (Thermo Fisher, Waltham, MA, USA). Global analysis of gene expression was performed with llumina HumanHT-12 v4 Bead Chip (Illumina, San Diego, CA, USA) consisting of more than 47 000 probes (ie. transcripts). For this purpose, TOP samples (n=1195 divided as follows: 358 CTRL, 388 SCH, 283 BD, and 166 MIX) passed labeling, hybridization and scanning. Raw microarray scan files were exported using the Illumina GenomeStudio software and loaded into R for downstream analysis using specific packages provided by BioConductor ^5^. Lumi ^6^ was used to detect outliers, which left 1111 samples. The sva ^7^ and ComBat packages ^8^ were used to correct for technical batch effects, like RNA extraction batch, RNA extraction method, DNase treatment batch, cRNA labelling batch and chip hybridization. Further quality control, quantile-normalization and log2-transformation was done using Limma ^9^. 1358 probes from chr16 were extracted based on TxDb.Hsapiens.UCSC.hg18.knownGene ^10^, R package version 3.2.3.

Data overlapping with individuals participating in the 16p11.2 distal imaging study was found for 235 non-carriers and 2 deletion carriers after removal of duplicate samples. Probes with a detection p-value below 0.99578, as defined by the detection value 0.99 in GenomeStudio and corrected for multiple testing (1-(0.01/237 individuals)), were considered not expressed (only background level expression) and not included in the analysis. This left 887 probes in the analysis. Mean and SD were calculated for both the deletion carrier group and the non-carrier group for each ProbeID in R, and the percentual difference was calculated as Mean(carriers)/Mean(non-carriers)*100. Only probes with more than 23 individuals above expression levels were kept (658 probes).

We focused on the expression of genes in a large interval 10 MB downstream and 3.3 MB downstream the 16p11.2 distal region including the 16p11.2 proximal region (chr16:18 400 000-32 500 000) (Supplementary Table 11) containing 226 probes. These 226 probes were plotted against the chromosomal coordinates of chr16:18 400 000-32 000 000 (hg18) with ggplot2 ^11^.

**Figure Legends, Supplementary Figures**

**Supplementary Figure 1: The coverage of the 16p11.2 region by the genotyping platforms in ENIGMA-CNV**. Log R ratio is shown in red, B-allele Frequency in blue. The vertical black lines delimit the boundaries of the 16p11.2 distal BP2-BP3 region. Affy, Human610, HumanOmni1S, OmniExpress, PsychChip and Exomechip are based on real data, the remaining is mock.

**Supplementary Figure 2: CNVs in the 16p11.2 distal region** in the ENIGMA-CNV sample as visualized in the UCSC Genome Browser. Start and end point as called by PennCNV. Duplications are colored blue and deletions red. All coordinates are in hg18.

**Supplementary Figure 3. Bivariate plot of age versus uncorrected caudate (a.), pallidum (b.), putamen (c.) at individual scanner sites.** Deletion carriers (del) in red, non-carriers (con) in grey and duplication carriers (dup) in blue, respectively.

**Supplementary Figure 4. Forest plots effect of change in 16p11.2 distal copy number on subcortical volumes, cortical surface area, cortical thickness and ICV.**

**a. Deletion vs duplication carriers. b. Deletion carriers vs non-carriers. c. Non-carriers vs duplication carriers.** The effect in Cohen’s D (difference in mean) at each site for each measure is shown by the position on the x-axis and the standard error is shown by the horizontal line. Analyses were corrected for age, age squared, sex, scanner site and ICV (except for ICV). A summary polygon shows the results when fitting a random-effects model to the two groups: ENIGMA-CNV discovery and deCODE replication samples. del, con and dup denote the number of individuals in each analysis. Pvalue is the p-value for each analysis where * = P < 0.005, ** = P < 0.0005. Effect size and confidence intervals are written on the right.

**Supplementary Figure 5: Full scale IQ in 16p11.2 distal carriers and non-carriers in a sample without psychiatric diagnoses.** Boxplots represent the mean of full scale IQ for four deletion carriers, 2 148 non-carriers and twelve duplication carriers. All comparisons between individual carrier groups are shown in Supplementary Table 8. Centre line represents median, box limits are the upper and lower 25 % quartiles, whiskers the 1.5 interquartile range, and the points are the outliers.

**Supplementary Figure 6: BMI z-scores in 16p11.2 distal carriers**. Deletion carriers are noted in red, non-carriers in grey and duplication carriers in blue, respectively. Centre line represents median, box limits are the upper and lower 25 % quartiles, whiskers the 1.5 interquartile range, and the points are the outliers.

**Supplementary Figure 7: Transcription in blood is decreased in deletion carriers of 16p11.2 distal in comparison to non-carriers.** Percentual mean difference in blood transcript of BP1-BP4 deletion carriers (n=2) and non-carriers (n=235) plotted against chromosomal position. The blue vertical lines indicates the core 16p11.2 distal region, the blue dashed lines the 16p11.2 distal extended region, and the grey dashed line the 16p11.2 proximal region. Only probes with more than 23 individuals above background detection in blood were included in the plot. **a.** Chr16:18 400 000-32 500 000 (166 probes) **b.** Close-up of the 16p11.2 distal and proximal region chr16:28 000 000-29 250 000.

**SUPPLEMENTARY TABLES**

**Supplementary Table 1: Specification of cohorts in ENIGMA CNV.** Please note that individuals from some chips have overlapping scanner sites. Data sets contributing data to the 16p11.2 distal analysis are marked in green.

**Supplementary Table 2: Extended information on 16p11.2 distal carriers.** Sex (1 = male, 2 = female), Established diagnosis (1 = yes, 0 = no), CN = copy number (1=deletion, 3=duplication). BMI z-scores are shown for individuals included in the mega-analysis. For those three carriers not available for mega-analysis (delcarrier 8, dupcarrier 6 and 9), BMI quantile within their own cohort is shown. Data on BMI for each cohort is shown in Supplementary Table 9. IQ in the form of FIQ (full scale IQ), verbal IQ (VIQ) and/or PIQ (performance IQ) is shown if known.

**Supplementary Table 3: CNVs of Interest.** Individuals with a minimum overlap of 0.4 to these CNVs were excluded from the analysis. Coordinates are Human Genome Build NCBI36/hg18.

| **CNV of interest** | **Chr** | **Start** | **Stop** | **Length** | **Risk for** | **Direction** |
| --- | --- | --- | --- | --- | --- | --- |
| 1q21.1 | 1 | 144 800 611 | 146 326 568 | 1 525 957 | Psych | risk |
| 2p16.3, NRXN1 | 2 | 50 558 547 | 51 518 397 | 959 850 | Psych | risk |
| 2p25.3, MYT1L | 2 | 1 733 617 | 2 204 151 | 470 534 | Psych | risk |
| 3q29 | 3 | 197 251 134 | 198 700 746 | 1 449 612 | Psych | risk |
| 7q11.21, ZNF92 | 7 | 6 4476 203 | 64 503 433 | 27 230 | SCZ | protect |
| 7q11.23 | 7 | 72 380 000 | 73 780 000 | 1 400 000 | SCZ | risk |
| 7q36.3, VIPR2 | 7 | 157 553 706 | 158 812 247 | 1 258 541 | Psych | risk |
| 8q22.2, VPS13B | 8 | 100 094 670 | 100 958 984 | 864 314 | SCZ | risk |
| 9p24.3, DMRT1 | 9 | 831 690 | 959 090 | 127 400 | SCZ | risk |
| 10q11.22-23 | 10 | 45 928 700 | 51 582 787 | 5 654 087 | Psych | risk |
| 13q12.11, ZMYM5 | 13 | 19 309 593 | 19 335 773 | 26 180 | SCZ | protect |
| 13q31.3, GPC6 | 13 | 93 156 194 | 93 217 895 | 61 701 | Psych | risk |
| 15q11.2 | 15 | 20 301 669 | 20 824 174 | 522 505 | Psych | risk |
| 15q11.2-13.1,BP1-2 | 15 | 20 322 358 | 26 208 861 | 5 886 503 | Psych | risk |
| 15q13.1,BP3-BP4 | 15 | 26 772 437 | 28 477 016 | 1 704 579 | Psych | risk |
| 15q13.3,BP4-BP5 | 15 | 28 723 577 | 30 303 141 | 1 579 564 | Psych | risk |
| 15q13.3,BP4.5-BP5 | 15 | 29 806 023 | 30 407 419 | 601 396 | ADHD | risk |
| 16p11.2, entire region | 16 | 28 351 599 | 30 100 062 | 1 748 463 | Psych | risk |
| 16p11.2 proximal | 16 | 29 502 984 | 30 100 062 | 597 078 | Psych | risk |
| 16p11.2 distal, large | 16 | 28 351 599 | 28 950 951 | 599 352 | Psych | risk |
| 16p11.2 distal | 16 | 28 721 599 | 28 950 951 | 229 352 | Psych | risk |
| 16p12.1 | 16 | 21 854 731 | 22 331 199 | 476 468 | Psych | risk |
| 16p13.11 | 16 | 14 897 345 | 16 199 484 | 1 302 139 | ADHD | risk |
| 17p12 | 17 | 14 041 754 | 15 411 904 | 1 370 150 | Psych | risk |
| 17q12 | 17 | 31 889 664 | 33 323 543 | 1 433 879 | Psych | risk |
| 22q11, 1Mb | 22 | 19 063 495 | 19 793 730 | 730 235 | Psych | risk |
| 22q11, 3Mb | 22 | 17 257 787 | 19 795 780 | 2 537 993 | Psych | risk, protect |
| Xq28 distal | X | 153 800 000 | 154 225 000 | 425 000 | SCZ | risk |
| Xq28, MAGEA11 | X | 14 8575 477 | 148 580 720 | 5 243 | SCZ | protect |

**Supplementary Table 4: Imaging Scanner parameters for the scanner sites in the 16p11.2 distal analysis.**

**Supplementary Table 5: Dose response of 16p11.2 distal copy number on subcortical volumes in ENIGMA-CNV.**  The effect size (β of the linear regression) is presented. A linear regression based on the copy number state of the individuals (deletion=1, normal=2, duplication=3) was performed on normalized brain measures corrected for *plusICV*: age, age squared, sex, scanner site and ICV (except for ICV) or *noICV*: age, age squared, sex, scanner site. Analysis was performed on either: ALL – all individuals, NON AFFECTED - excluding individuals with established diagnosis, ADULTS - including individuals above 18 years or MATCHED CONTROLS - matching each carrier with four non-carriers based on age, gender, presence/absence of established diagnosis. Analysis was performed INCLUDING RELATIVES (left table) or EXCLUDING RELATIVES (right table) – relatives being defined as more than third degree relationships. Results were considered statistically significant if they were below a Bonferroni-corrected P-value of 0.005 (0.05/10 regions). * = P < 0.005, ** = P < 0.0005.

**Supplementary Table 6: T-test on subcortical volumes between different 16p11.2 distal copy number groups.** The effect size (Cohen’s D) is presented. Deletion versus duplication carriers, deletion carriers versus non-carriers and non-carriers versus duplication carriers in ENIGMA (discovery). T-tests were performed on normalized values of brain measures corrected for *plusICV*: age, age squared, sex, scanner site and ICV (except for ICV). ALL – all individuals, NON AFFECTED - excluding individuals with established diagnosis, ADULTS - including individuals above 18 years or MATCHED CONTROLS - matching each carrier with four non-carriers based on age, gender, presence/absence of established diagnosis. Analysis was performed INCLUDING RELATIVES (left table) or EXCLUDING RELATIVES (right table) – relatives being defined as more than third degree relationships. Results were considered statistically significant if they were below a Bonferroni-corrected P-value of 0.005 (0.05/10 regions). * = P < 0.005, ** = P < 0.0005.

**Supplementary Table 7: Check of effect of population stratification: Dose response of 16p11.2 distal copy number on subcortical volumes with and without correcting for ancestry.** A linear regression based on the copy number state of the individuals (deletion=1, normal=2, duplication=3) was performed on normalized brain measures corrected for **A**. age, age squared, sex, scanner site and ICV (except for ICV) or **B**. age, age squared, sex, scanner site and ICV (except for ICV), C1, C2, C3 and C4. Only individuals with accessible ancestry information were included in the analysis. The effect size (β of the linear regression) is presented.

**Supplementary Table 8: Difference in full scale IQ in 16p11.2 distal carriers**. A Kruskal-Wallis test was performed for the deCODE chort. Comparison between individual carrier groups (deletion (del), duplication (dup) and non-carriers (con)) was tested with Wilcoxon rank sum test with continuity. Results were considered significant at a cutoff of P<0.05.

|  |  |  |  |  | **Wilcoxon** | |  | **Kruskal-Wallis** | | |
| --- | --- | --- | --- | --- | --- | --- | --- | --- | --- | --- |
| **CN1** | **CN2** | **n, CN1** | **n, CN2** |  | **W** | **P** |  | **chi square** | **df** | **P** |
| del | con | 4 | 2148 |  | 1 133 | 0.011 |  | 10.9 | 2 | 0.0042 |
| con | dup | 2 148 | 12 |  | 17 432 | 0.035 |  |  |  |  |
| del | dup | 4 | 12 |  |  |  |  |  |  |  |

**Supplementary Table 9: Summary of BMI in each cohort.** Cohorts not available for mega-analysis are marked with *.

| **Cohort** | **del** | | **con** | **dup** | |  | **Total n** | **Mean** | **Sd** | **Median** | **Min** | **Max** |
| --- | --- | --- | --- | --- | --- | --- | --- | --- | --- | --- | --- | --- |
| ***Mega-analysis*** | | |  |  | |  |  |  |  |  |  |  |
| 16p11.2 Consortium | 4 | | 32 | 3 | |  | 39 | 24 | 5.7 | 23.1 | 15 | 41 |
| GOBS | 1 | | 390 |  | |  | 391 | 30.8 | 6.8 | 30.3 | 16 | 49 |
| HUNT |  | | 873 | 1 | |  | 874 | 26.9 | 3.7 | 26.6 | 18 | 42 |
| Imagen |  | | 20 | 2 | |  | 22 | 20.1 | 3.2 | 19.1 | 16 | 26 |
| OATS |  | | 256 | 1 | |  | 257 | 27.1 | 4.4 | 26.0 | 16 | 45 |
| TOP | 2 | | 309 |  | |  | 311 | 25.7 | 4.6 | 25.4 | 15 | 41 |
| Total | 7 | | 1 880 | 7 | |  |  |  |  |  |  |  |
|  |  | |  |  | |  |  |  |  |  |  |  |
| ***Reports on single cohorts*** | | | | | |  |  |  |  |  |  |  |
| Jülich* | | 1 | 744 |  |  |  | 745 | 27.4 | 4.1 | 26.9 | 19 | 44 |
| SHIP* | |  | 1 842 | | 2 |  | 1844 | 27.5 | 4.4 | 27.0 | 17 | 48 |

**Supplementary Table 10: Difference in BMI z-scores in 16p11.2 distal carriers.** A Kruskal-Wallis test was performed. Comparison between individual carrier groups (deletion (del), duplication (dup) and non-carrier controls (con)) was tested with Wilcoxon rank sum test with continuity correction. Results were considered significant at a cutoff of P<0.05.

|  |  |  |  |  | **Wilcoxon** | |  | **Kruskal-Wallis** | | |
| --- | --- | --- | --- | --- | --- | --- | --- | --- | --- | --- |
| **CN1** | **CN2** | **n, CN1** | **n, CN2** |  | **W** | **P** |  | **chi square** | **df** | **P** |
| del | con | 7 | 1 880 |  | 9 290 | 0.18 |  | 9.4 | 2 | 0.0092 |
| con | dup | 1 880 | 7 |  | 3 109 | 0.048 |  |  |  |  |
| del | dup | 7 | 7 |  | 43 | 0.052 |  |  |  |  |

**Supplementary Table 11: Blood gene Expression levels in two 16p11.2 distal (BP1-BP4) carriers versus 235 non-carriers.** The core 16p11.2 distal region is marked in dark grey, the extended region in light gray. Probetype: A=All isoforms, I=isoform specific, S=single isoform, M=multiple isoforms. Pvalueabovecutoff indicates the number of individuals with detection Pvalue above 0.99578 in the analysis. Probes with less than 24 individuals above the 0.99578 Pvalue detection limit were excluded in the visualization in Figures 2A and 2B. Pvalue_carrier1 and Pvalue_carrier2 indicate the Pvalue for detection for each deletion carrier (Delcarrier1 and delcarrier2)

1. Higgins JP, Thompson SG, Deeks JJ, Altman DG. Measuring inconsistency in meta-analyses. *BMJ (Clinical research ed)* 2003; **327**(7414)**:** 557-560.

2. Wechsler D. *Wechsler Abbreviated Scale of Intelligence*. Harcourt Brace and Company1999.

3. Gudmundsson E. *Mat á greind fullorðinna: WASIIS [The Assessment of Intelligence in Adults: WASIIS].* . Menntamalastofnun, Reykjavik, Iceland2015.

4. Wechsler D. *Wechsler Adult Intelligence Scale* The Psychological Corporation, San Antonio, TX1997.

5. Ritchie ME, Dunning MJ, Smith ML, Shi W, Lynch AG. BeadArray expression analysis using bioconductor. *PLoS computational biology* 2011; **7**(12)**:** e1002276.

6. Du P, Kibbe WA, Lin SM. lumi: a pipeline for processing Illumina microarray. *Bioinformatics (Oxford, England)* 2008; **24**(13)**:** 1547-1548.

7. Leek JT JW, Parker HS, Fertig EJ, Jaffe AE, Storey JD, Zhang Y, Torres LC sva: Surrogate Variable Analysis. *R package version 3244* 2017.

8. Johnson WE, Li C, Rabinovic A. Adjusting batch effects in microarray expression data using empirical Bayes methods. *Biostatistics (Oxford, England)* 2007; **8**(1)**:** 118-127.

9. Ritchie ME, Phipson B, Wu D, Hu Y, Law CW, Shi W *et al.* limma powers differential expression analyses for RNA-sequencing and microarray studies. *Nucleic acids research* 2015; **43**(7)**:** e47.

10. Maintainer MCaBP. TxDb.Hsapiens.UCSC.hg18.knownGene: Annotation package for TxDb object(s). *R package version 322* 2015.

11. Wickham H. *ggplot2: Elegant Graphics for Data Analysis.* Springer-Verlag: New Yrok, 2009.
