# Supplementary material for: Dose response of the 16p11.2 distal copy number variant on intracranial volume and basal ganglia
Source: Mol Psychiatry. 2018 Oct 3;25(3):584–602. doi: 10.1038/s41380-018-0118-1 (PMC7042770; doi:10.1038/s41380-018-0118-1)

Supplementary Figure 1

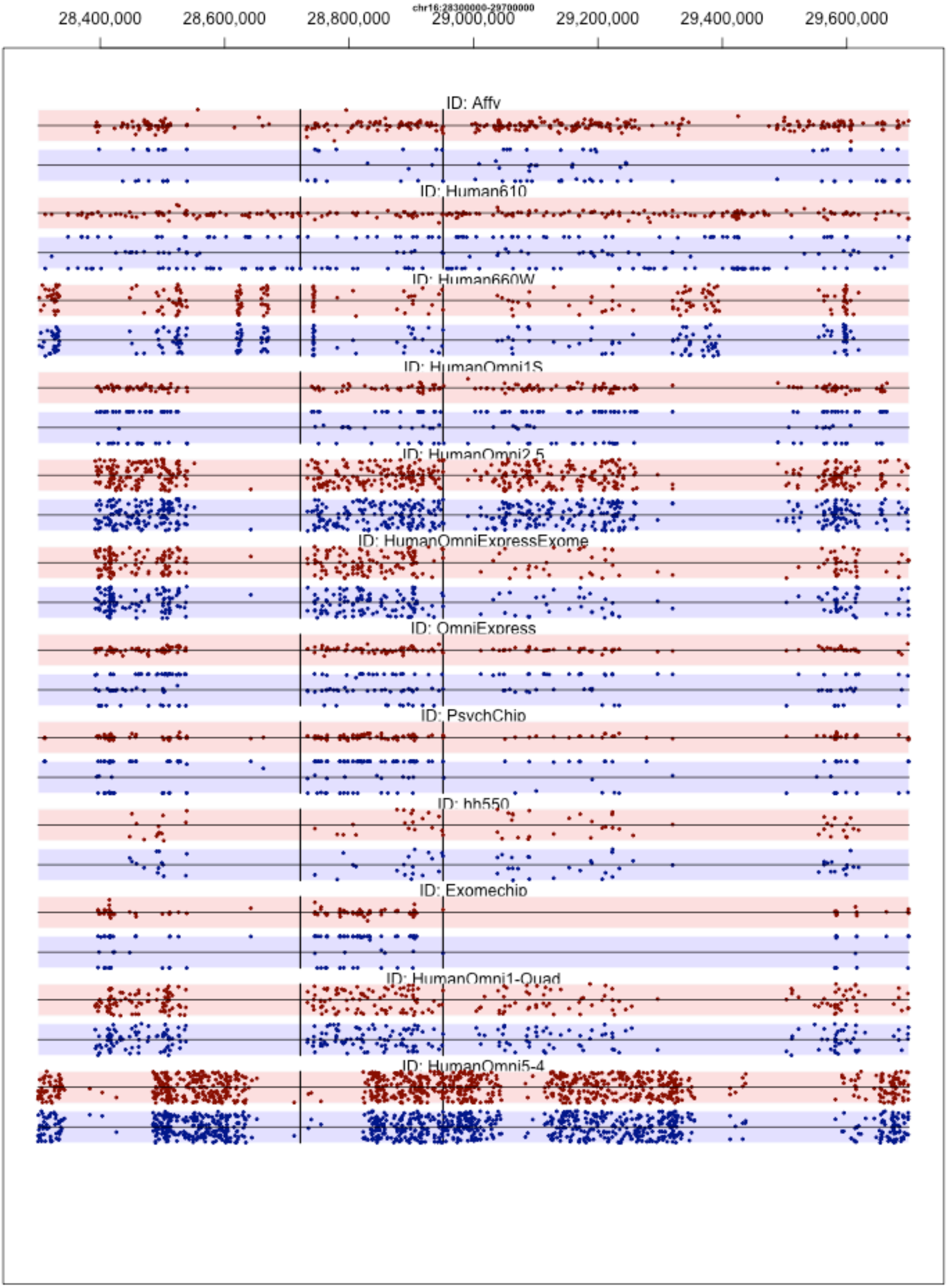

Supplementary Figure 2

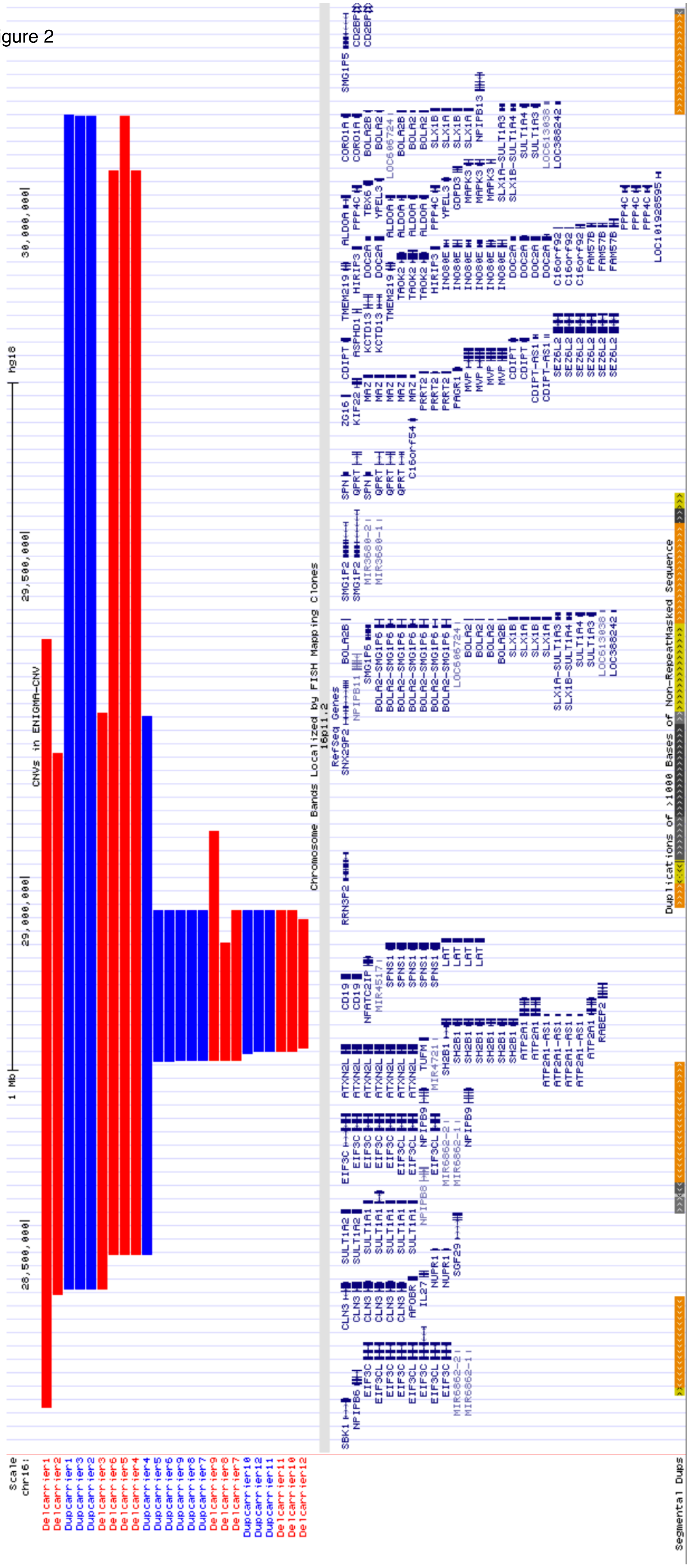

Supplementary Figure 3a

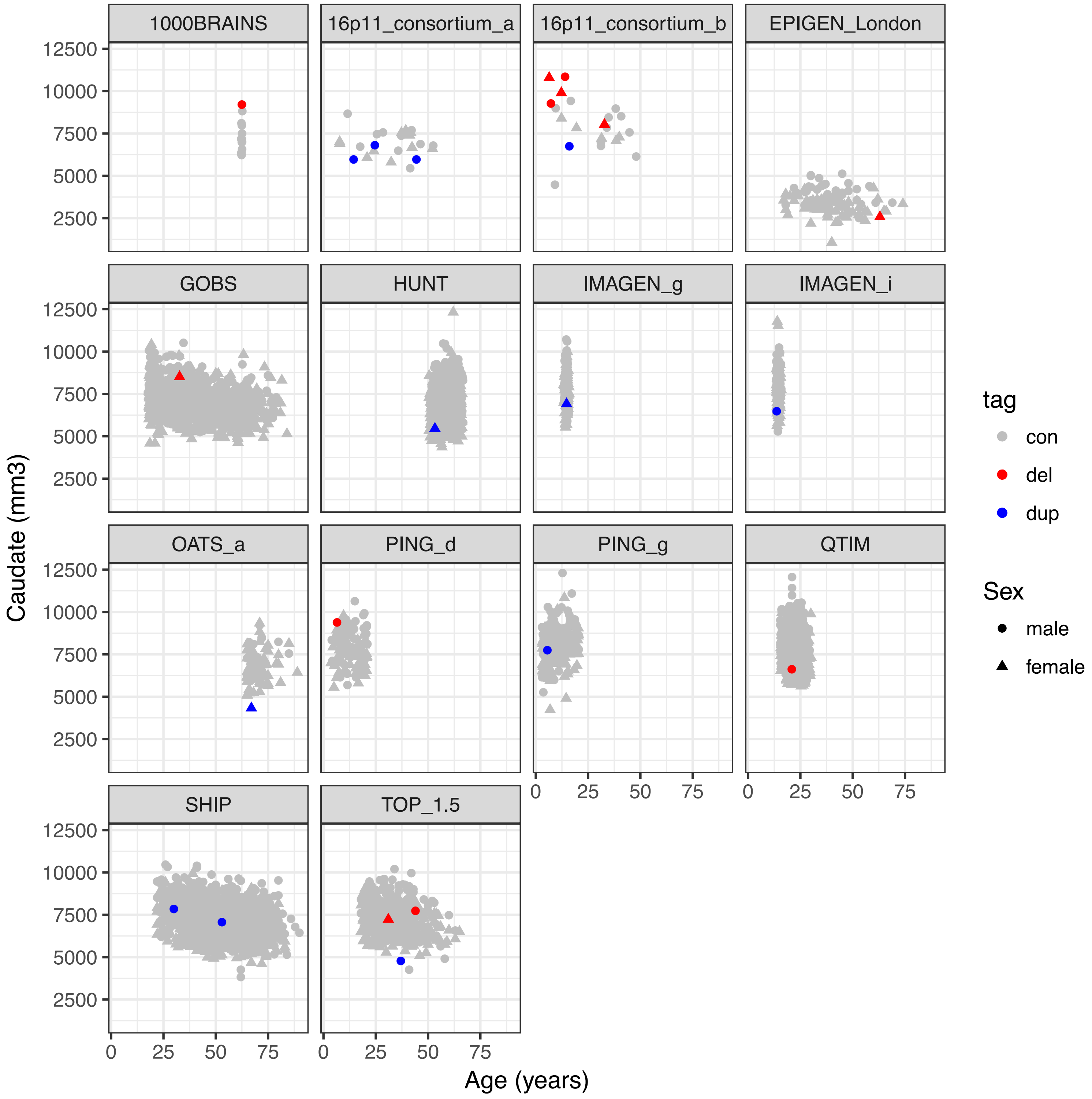

Supplementary Figure 3b

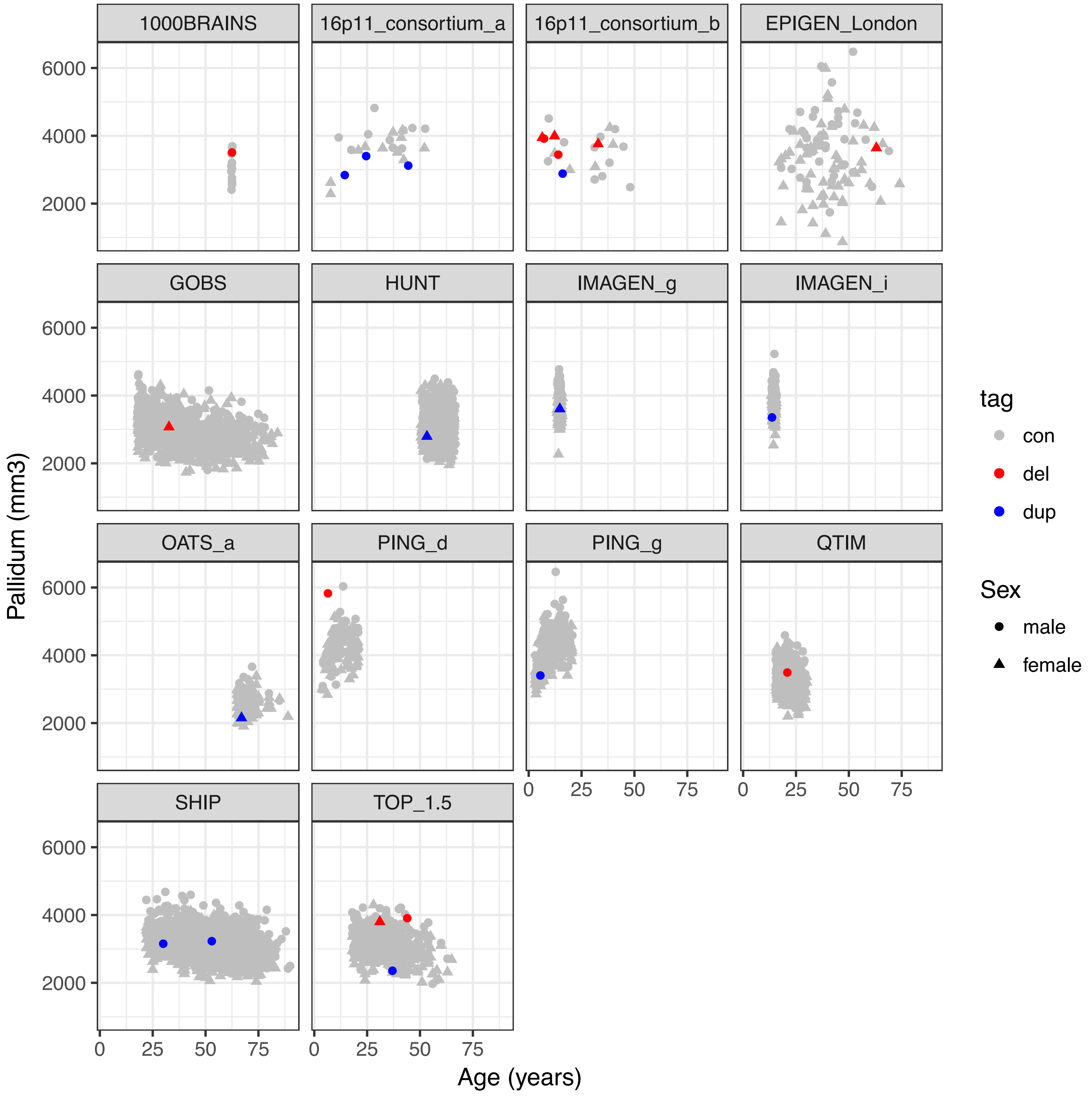

Supplementary Figure 3c

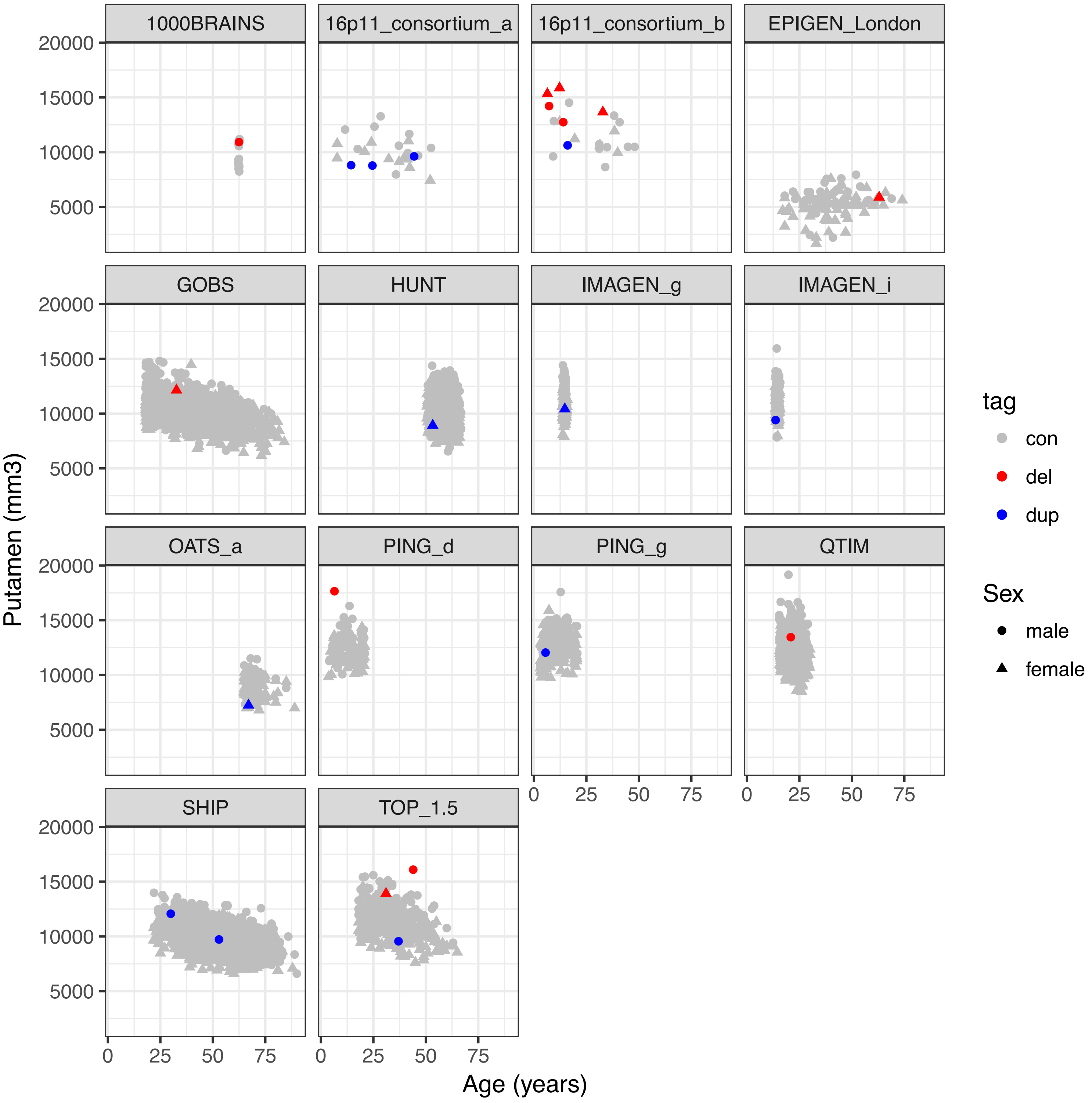

Supplementary Figure 4a

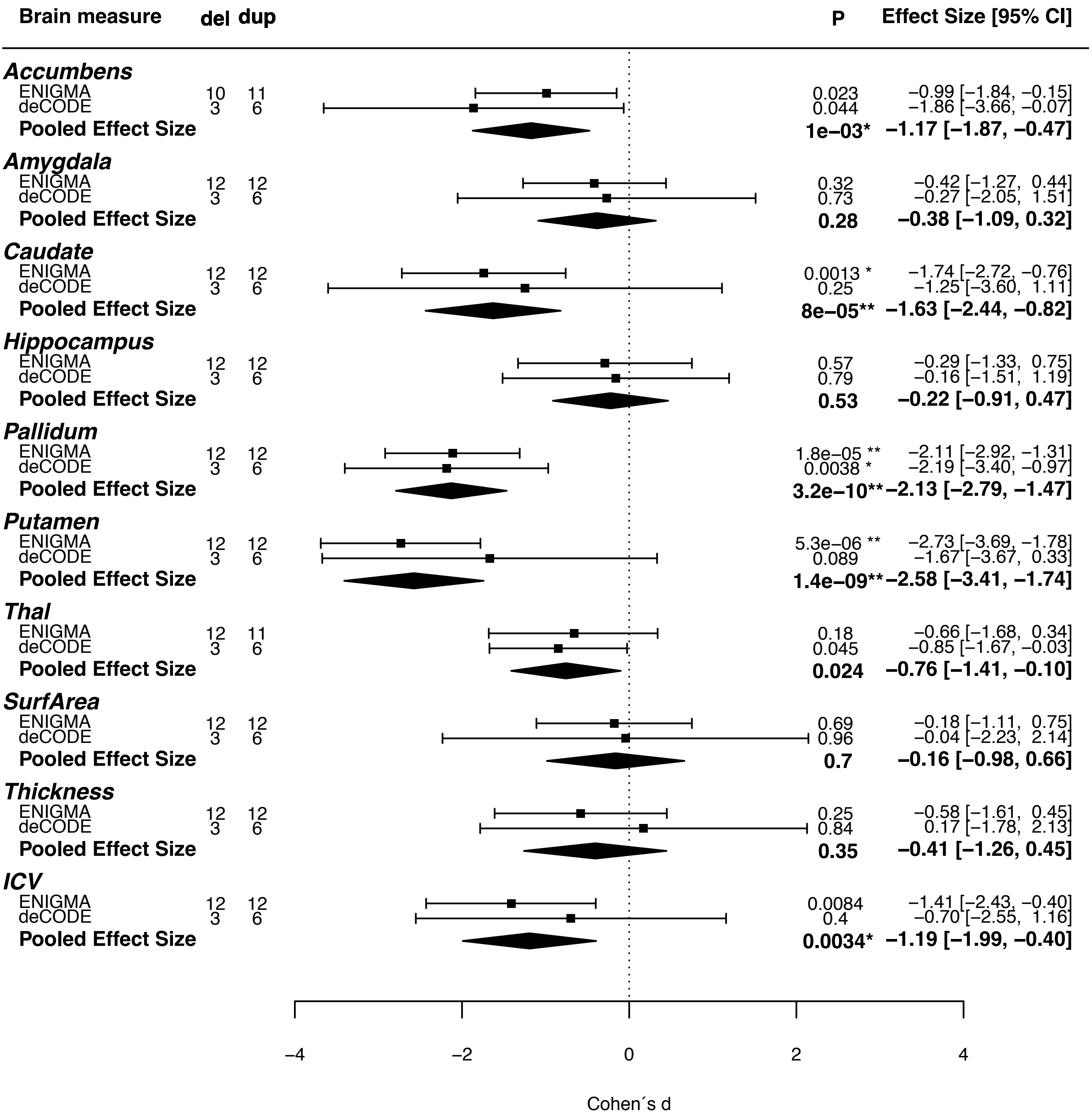

Supplementary Figure 4b

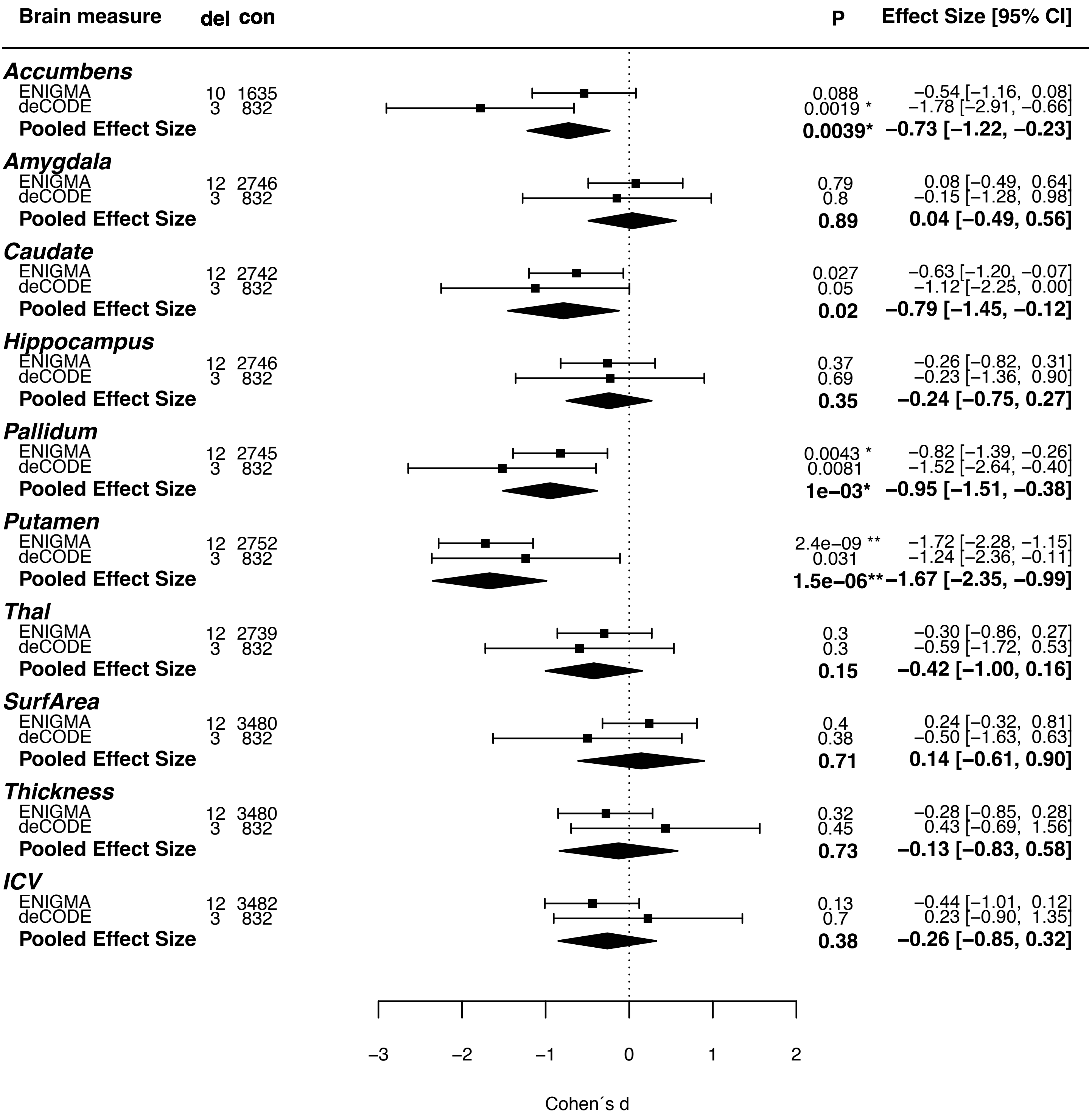

Supplementary Figure 4c

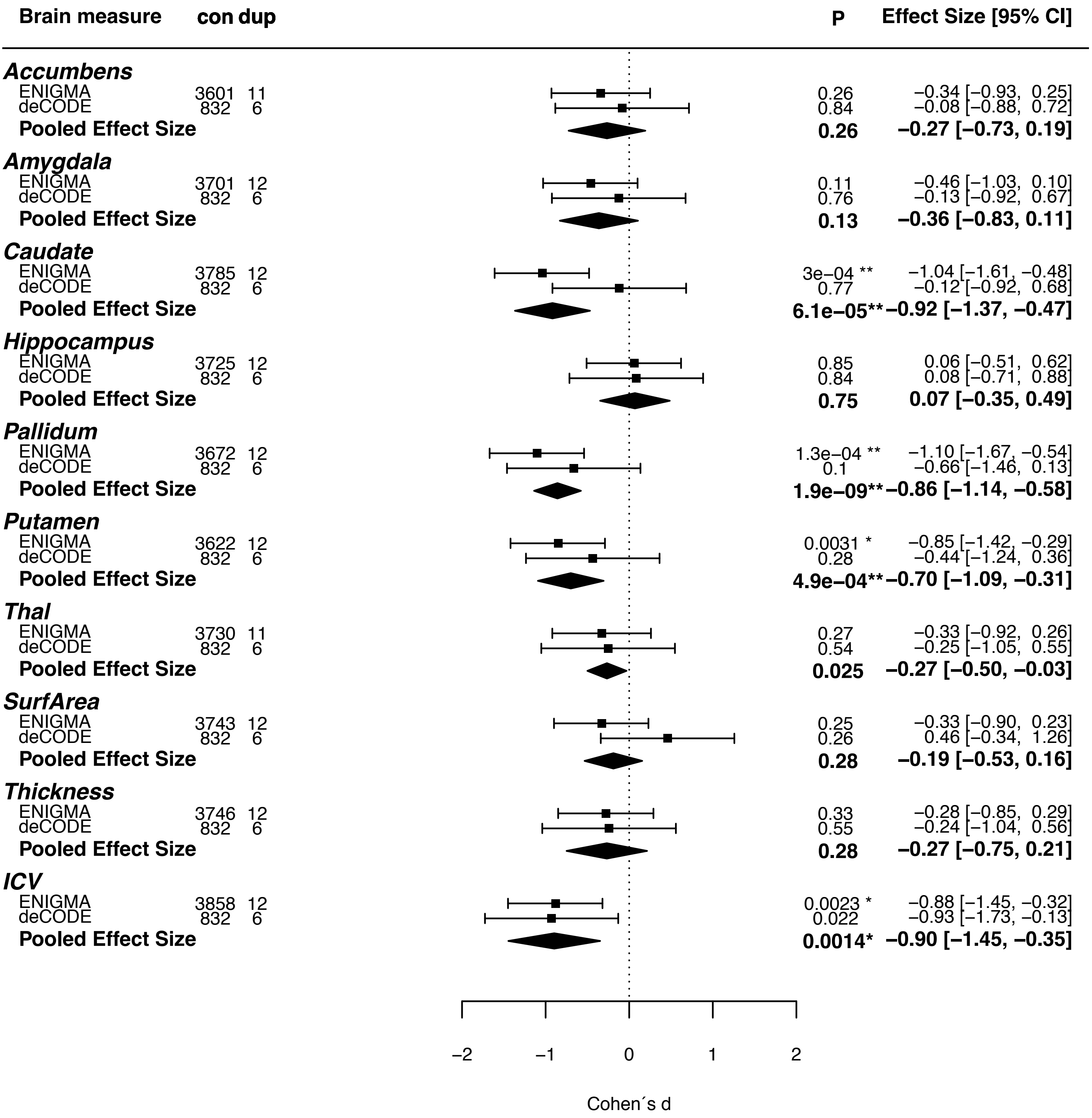

Supplementary Figure 5

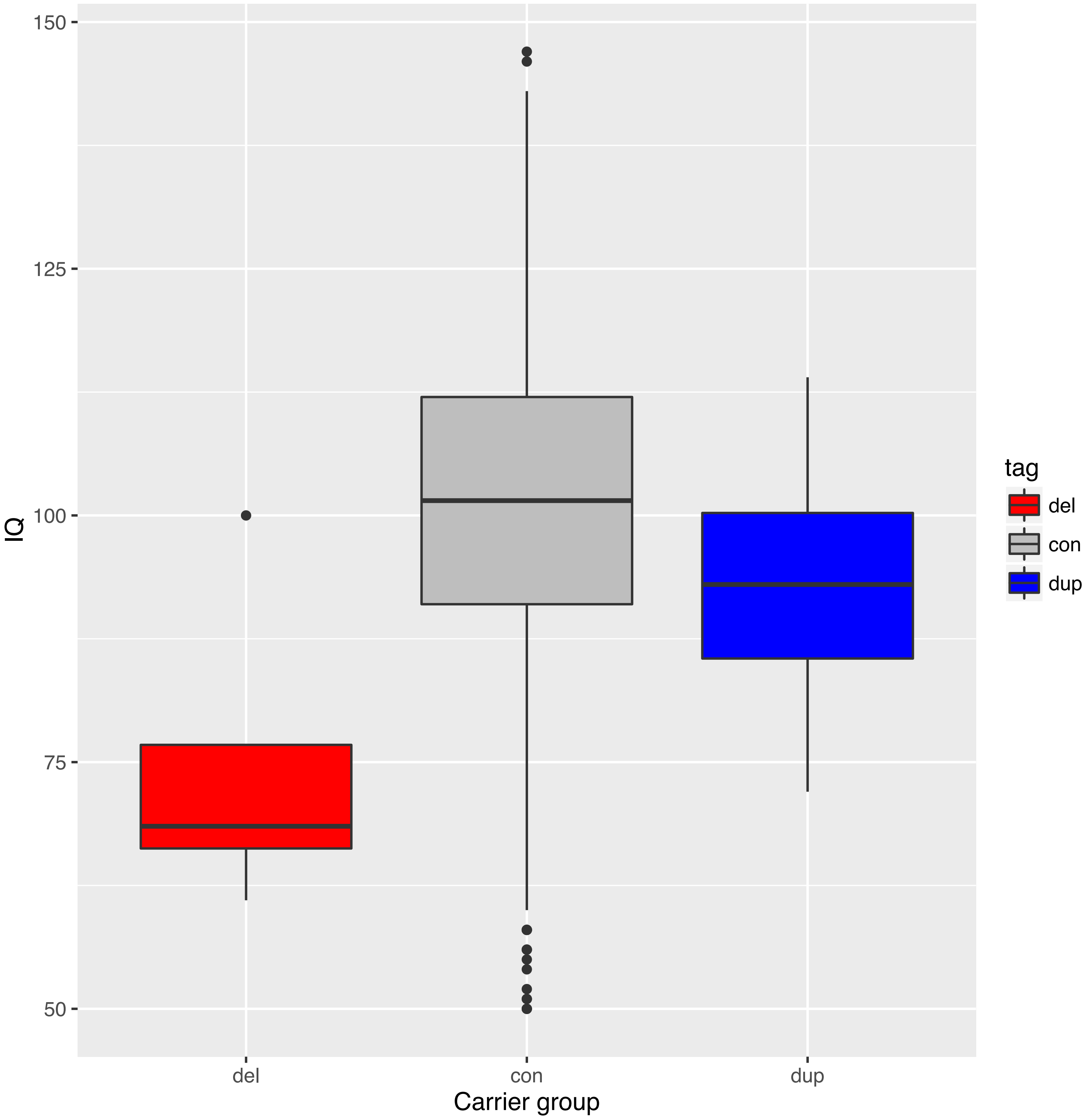

Supplementary Figure 6

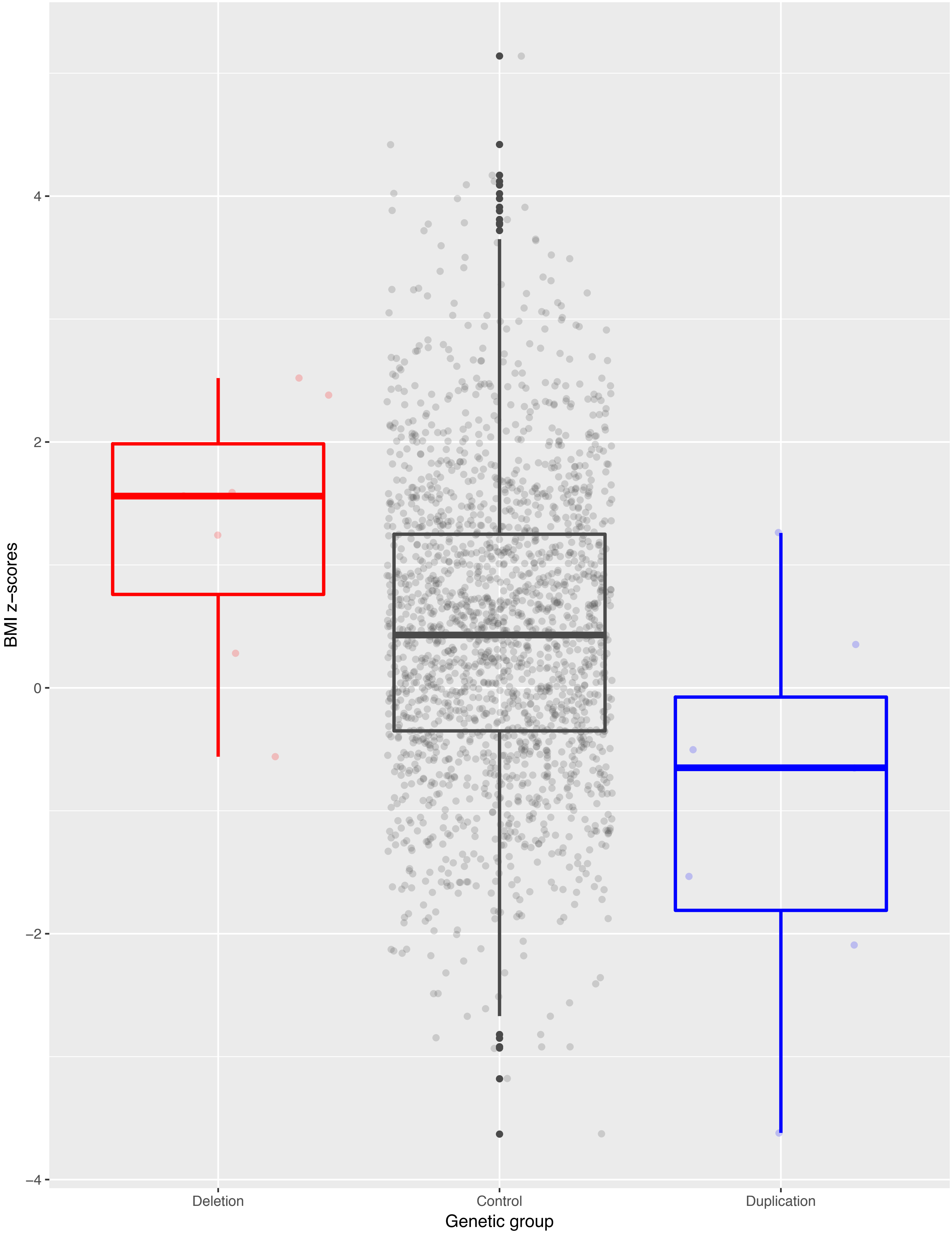

Supplementary Figure 7

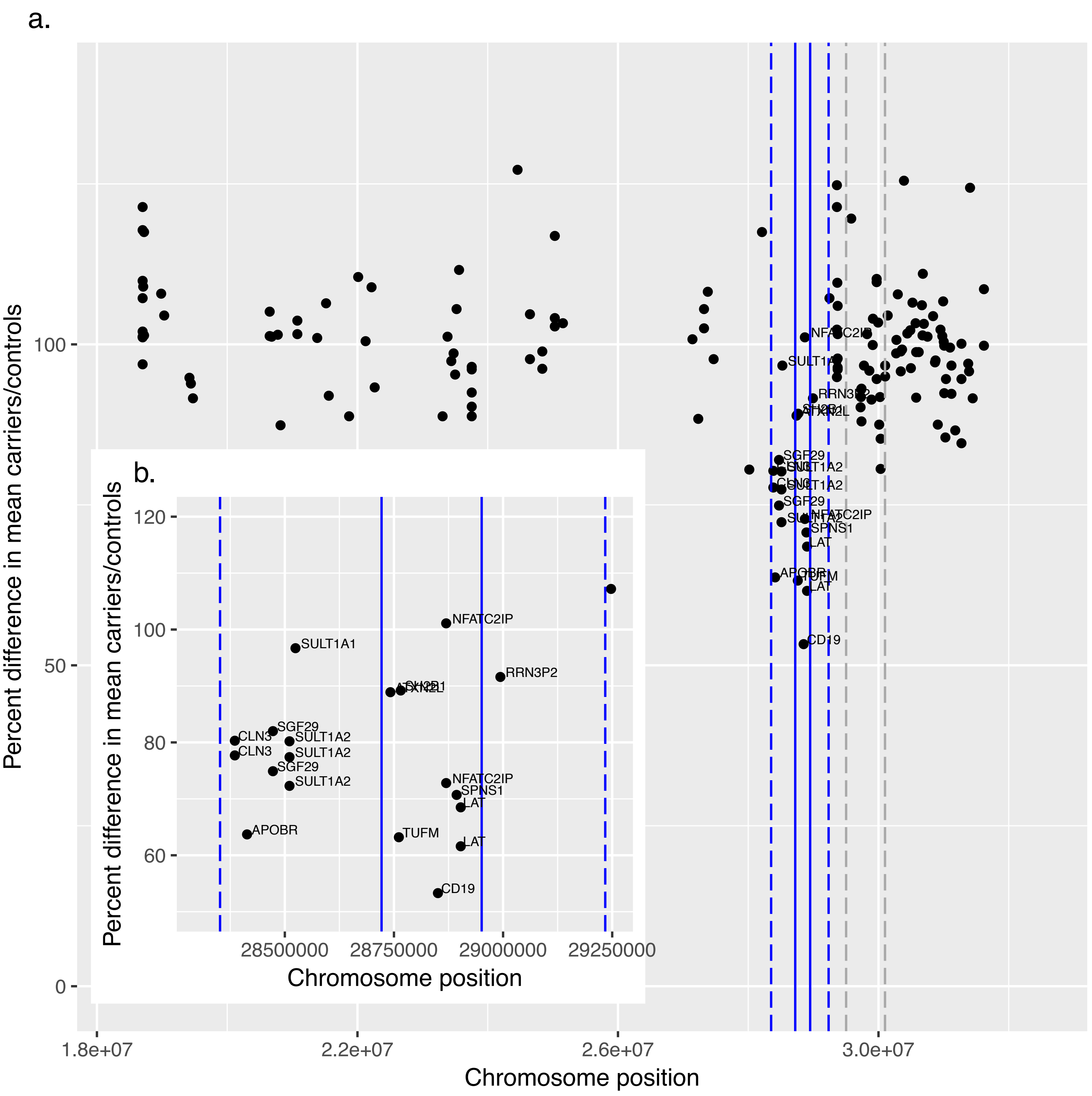

Supplement: Supplementary file 2 — Supplementary figures [file 41380_2018_118_MOESM2_ESM.pdf]
